# g-C_3_N_4_ Modified with Metal Sulfides for Visible-Light-Driven Photocatalytic Degradation of Organic Pollutants

**DOI:** 10.3390/molecules30020253

**Published:** 2025-01-10

**Authors:** Shoaib Mukhtar, Erzsébet Szabó-Bárdos, Csilla Őze, Tatjána Juzsakova, Kornél Rácz, Miklós Németh, Ottó Horváth

**Affiliations:** 1 Research Group of Environmental and Inorganic Photochemistry, Center for Natural Sciences, Faculty of Engineering, University of Pannonia, P.O. Box 158, H-8201 Veszprém, Hungary; mukhtar.shoaib@phd.mk.uni-pannon.hu (S.M.); szabone.bardos.erzsebet@mk.uni-pannon.hu (E.S.-B.); 2 Department of Materials Engineering, Research Center for Engineering Sciences, University of Pannonia, P.O. Box 158, H-8201 Veszprém, Hungary; oze.csilla@mk.uni-pannon.hu; 3 Sustainability Solutions Research Lab, Research Center for Biochemical, Environmental and Chemical Engineering, University of Pannonia, P.O. Box 158, H-8201 Veszprém, Hungary; juzsakova.tatjana@mk.uni-pannon.hu; 4 Nanolab, Research Institute of Biomolecular and Chemical Engineering, University of Pannonia, P.O. Box 158, H-8201 Veszprém, Hungary; racz.kornel@mk.uni-pannon.hu; 5 HUN-REN–PE Environmental Mineralogy Research Group, P.O. Box 158, H-8201 Veszprém, Hungary; 6 Centre for Energy Research, Surface Chemistry and Catalysis Department, Konkoly-Thege Street 29–33, H-1121 Budapest, Hungary; nemeth.miklos@ek-cer.hu

**Keywords:** g-C_3_N_4_-Bi_2_S_3_ nanocomposite, g-C_3_N_4_-ZnS nanocomposite, starch-assisted synthesis, visible-light-driven photocatalyst, coumarin, para-nitrophenol

## Abstract

Graphitic carbon nitride (g-C_3_N_4_) proved to be a promising semiconductor for the photocatalytic degradation of various organic pollutants. However, its efficacy is limited by a fast electron hole recombination, a restricted quantity of active sites, and a modest absorption in the visible range. To overcome these limitations, g-C_3_N_4_-Bi_2_S_3_ and g-C_3_N_4_-ZnS composites were effectively produced utilizing a starch-assisted technique. The findings from FT-IR, XRD, EDX, XPS, BET, SEM, and TEM demonstrated that the enhanced photocatalytic activity of g-C_3_N_4_-Bi_2_S_3_ and g-C_3_N_4_-ZnS composites was primarily due to their improved photocarrier separation and transfer rates. The photocatalyst facilitated the aerobic photocatalytic degradation of colorless contaminants such as coumarin and para-nitrophenol (4-NP). For the decomposition of 4-NP, g-C_3_N_4_-Bi_2_S_3_ exhibited a maximum efficiency of 90.86% in UV light and 16.78% in visible light, with rate constants of 0.29 h^−^^1^ and 0.016 h^−^^1^, respectively. In contrast, g-C_3_N_4_-ZnS demonstrated a maximum efficiency of 100% in UV light and 15.1% in visible light, with rate constants of 0.57 h^−^^1^ and 0.018 h^−^^1^, respectively. The bioinspired synthesis combined with the modification with metal sulfides proved to considerably enhance the photocatalytic activity of g-C_3_N_4_, increasing its potential for practical applicability in environmentally friendly water treatment systems for the efficient removal of recalcitrant organic contaminants.

## 1. Introduction

Water pollution from organic pollutants has emerged as a significant worldwide issue owing to the release of toxic compounds from industrial and agricultural sources [1]. Two notably colorless contaminants include coumarin [2] and para-nitrophenol [3]. Coumarin, extensively used in the pharmaceutical and fragrance sectors [4], and para-nitrophenol, often found in dyes, insecticides, and pharmaceuticals [5], are both recognized for their long lifespans and toxicity, making them challenging to decompose in the environment. Traditional wastewater treatment techniques frequently fail to succeed in completely decomposing these intricate compounds, resulting in prolonged environmental and health issues [6]. In response, researchers are progressively investigating photocatalysis as a sustainable method that harnesses solar energy to facilitate reactions that can break down persistent organic pollutants [7]. Photocatalysis entails the activation of a catalyst using light energy, resulting in the generation of reactive electrons and holes capable of degrading contaminants [8]. An efficient photocatalyst must exhibit responsiveness to visible light, stability, and cost-effectiveness. Graphitic carbon nitride (g-C_3_N_4_) has potential as a photocatalyst owing to its stability, reactivity to visible light, and cost-effective production. Nonetheless, g-C_3_N_4_ has inherent limitations, such as a propensity for fast electron hole recombination and a restricted quantity of active sites, which diminish its overall efficacy. Researchers have produced composite materials that combine g-C_3_N_4_ with other semiconductors to boost performance by improving charge separation, expanding light absorption, and increasing catalytic activity [9].

Two fascinating metal sulfides for coupling with g-C_3_N_4_ are bismuth sulfide (Bi_2_S_3_) and zinc sulfide (ZnS). Bi_2_S_3_, characterized by its small band gap and robust visible-light absorption [10], enhances the light-harvesting efficiency of g-C_3_N_4_, while ZnS, with advantageous band alignment and exceptional stability, promotes electron transfer and prolongs the longevity of charge carriers [11]. Bismuth sulfide (Bi_2_S_3_) [12] and zinc sulfide (ZnS) [13] are excellent semiconductors for photocatalytic applications, each possessing unique benefits. Bi_2_S_3_, with a small band gap of 1.3–1.7 eV, effectively absorbs visible light and creates reactive oxygen species (e.g., ^•^OH, O_2_^•−^), rendering it proficient in the degradation of organic pollutants [14], hydrogen evolution [15], and CO_2_ reduction [16]. Nonetheless, its photocatalytic efficacy is limited by photodegradation and charge recombination, which may be alleviated by creating heterojunctions with materials like g-C_3_N_4_. Conversely, ZnS, a wide-band-gap semiconductor (~3.6 eV), has superior performance in UV light-driven photocatalysis owing to its photostability and elevated charge mobility; yet it is limited by inadequate visible light absorption and elevated carrier recombination rates [17]. However, it can be applied in composites to decrease charge recombination, e.g., in visible-light-driven H_2_ production [18]. The amalgamation of these materials utilizes the visible-light activity of Bi_2_S_3_ and the stability of ZnS, hence improving charge separation and expanding the light absorption spectrum, making the composite an efficient system for environmental remediation.

The formation of g-C_3_N_4_-Bi_2_S_3_ [19] and g-C_3_N_4_-ZnS [20] composites enables these materials to synergistically improve photocatalytic efficiency under visible light. It is anticipated that these composites would outperform pure g-C_3_N_4_ in degrading colorless contaminants such as para-nitrophenol and coumarin, providing a more potent remedy for environmental remediation [21]. Using starch as a sacrificial template, this work synthesizes g-C_3_N_4_-Bi_2_S_3_ and g-C_3_N_4_-ZnS composites using a simple, low-temperature synthesis technique. By helping to evenly distribute Bi_2_S_3_ and ZnS on the g-C_3_N_4_ surface, the starch provides an economical and environmentally friendly method of producing these nanocomposites [22].

The resultant photocatalysts exhibited enhanced UV and visible-light efficacy owing to superior charge separation, broader light absorption, and more active sites. We analyze their efficacy in degrading coumarin and para-nitrophenol under UV and visible light, which acts as a model reaction to evaluate their photocatalytic properties. For the interpretation of the results, the band-gap energies and positions were taken into consideration. This study emphasizes the efficacy of g-C_3_N_4_-Bi_2_S_3_ and g-C_3_N_4_-ZnS as photocatalysts for environmental remediation, contributing significant insights to sustainable wastewater treatment.

Unlike prior works that produced Bi_2_S_3_ and ZnS using starch or applied hydrothermal techniques for g-C_3_N_4_-Bi_2_S_3_ and g-C_3_N_4_-ZnS composites, to our knowledge this study gives the first report of a starch-modified g-C_3_N_4_-Bi_2_S_3_ and g-C_3_N_4_-ZnS composite for photocatalytic applications. Furthermore, while many studies focus on the degradation of colored dyes, which are relatively easy to degrade due to their visible-light absorption, this work investigates the degradation of nitrophenol, a more persistent and environmentally relevant pollutant. The findings emphasize the potential of starch as a sustainable modification for ZnS composites, presenting a new way to boost photocatalytic performance for challenging pollutant systems.

## 2. Results and Discussion

Graphitic carbon nitride has been modified with various amounts of Bi_2_S_3_ and ZnS, as it is described in Section 3.3. within the experimental part. The corresponding catalyst samples were designated as g-C_3_N_4_-Bi_2_S_3_(1), g-C_3_N_4_-Bi_2_S_3_(2), and g-C_3_N_4_-Bi_2_S_3_(3) as well as g-C_3_N_4_-ZnS(1), g-C_3_N_4_-ZnS(2), and g-C_3_N_4_-ZnS(3). Their Bi, Zn, and S contents were measured by ICP, and the results are summarized in the Supplementary Materials (SM, Table S1). According to the corresponding data, a considerable part of Bi and a smaller fraction of Zn existed in the samples as oxides instead of sulfides. It is the consequence of the aerated preparation method.

### 2.1. FTIR Analysis

**Figure 1:**
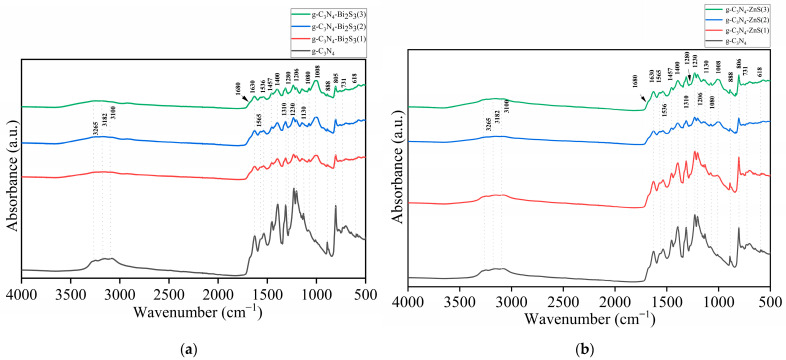
FTIR spectra of (**a**) g-C_3_N_4_ and g-C_3_N_4_-ZnS; (**b**) g-C_3_N_4_, and g-C_3_N_4_-Bi_2_S_3_.

The vibrational modes of the material triazine (C_3_N_3_) ring structure are shown by a number of distinctive peaks in the FTIR spectrum of pure g-C_3_N_4_ (Figure 1a,b black curve). Strong covalent bonds are present in the heptazine units that make up the framework of g-C_3_N_4_, as seen by the most noticeable peak at 1630 cm^−^^1^, which is ascribed to the C=N stretching vibrations. The graphitic carbon nitride structure [23] is further confirmed by peaks between 1206 cm^−^^1^ and 1310 cm^−^^1^, which correspond to C-N stretching vibrations inside the aromatic rings. The out-of-plane bending of the triazine units, a fingerprint characteristic for g-C_3_N_4_, is shown by the peak at 805 cm^−^^1^, which makes it especially significant [24]. N-H or O-H stretching vibrations are indicated by the large bands about 3000–3500 cm^−^^1^, which are probably caused by adsorbed moisture or surface amine groups [25].

The FTIR spectra in Figure 1a,b show significant changes when Bi_2_S_3_ and ZnS are introduced to g-C_3_N_4_, as shown by the green g-C_3_N_4_-Bi_2_S_3_(3) and g-C_3_N_4_-ZnS(3), blue g-C_3_N_4_-Bi_2_S_3_(2) and g-C_3_N_4_-ZnS(2), and red g-C_3_N_4_-Bi_2_S_3_(1) and g-C_3_N_4_-ZnS(1) colors. Especially at higher concentrations, the expansion of the peak at 1008 cm^−^^1^, which correlates to C-N stretching vibrations in the g-C_3_N_4_ framework, is the most notable alteration. Localized strain or flaws in the g-C_3_N_4_ structure brought on by the physical interaction of Bi_2_S_3_ and ZnS with the surface are probably the origin of this widening. A larger variety of vibrational frequencies might also occur from a shift in electron density around the C-N bonds caused by charge transfer effects between the semiconducting Bi_2_S_3_, ZnS, and g-C_3_N_4_. Similar widening is also seen in the peaks in the 1206–1310 cm^−^^1^ range, suggesting that Bi_2_S_3_ and ZnS influence the local vibrational environment without significantly changing the g-C_3_N_4_ framework.

Notwithstanding these modifications, the fundamental structure of g-C_3_N_4_ remains mostly unaltered after the incorporation of both sulfides, as seen by the persistence of the 805 cm^−^^1^ peak, which remains distinct and in the same location. The widening of additional peaks and the little decrease in intensity indicate that the connection between g-C_3_N_4_ and Bi_2_S_3_ as well as g-C_3_N_4_ and ZnS, is mostly physical or mediated by weak van der Waals forces, rather than strong covalent bonding. The absence of additional peaks in the spectra corroborates this, indicating that no new chemical bonds are established between the two components. The introduction of Bi_2_S_3_ and ZnS likely creates surface heterogeneity and alters the electrical environment of the g-C_3_N_4_ surface, resulting in minor variations in the FTIR spectrum while preserving the fundamental chemical structure. For comparison, the FTIR spectra of Bi_2_S_3_ and ZnS are provided as Figure S1.

The essential role of starch in the modification process probably provides supplementary hydroxyl groups or other functional groups that may modify the hydrogen bonding or other weak interactions between g-C_3_N_4_ and metal sulfides. This may explain the nuanced variations and the expansion of the peaks without significant changes in the chemical structure, as shown in the spectrum. The persistence of the 805 cm^−^^1^ signal indicates that the core structure of g-C_3_N_4_ is stable despite the change [26]. The O-H stretching vibrations from starch may also contribute to the wide bands between 3000 and 3500 cm^−^^1^, reinforcing the idea that starch induces surface changes and influences the overall bonding environment in the composite material [27]. This surface modification improves dispersion, stability, and interactions within the composite, resulting in observed spectrum alterations.

### 2.2. XRD Analysis

**Figure 2:**
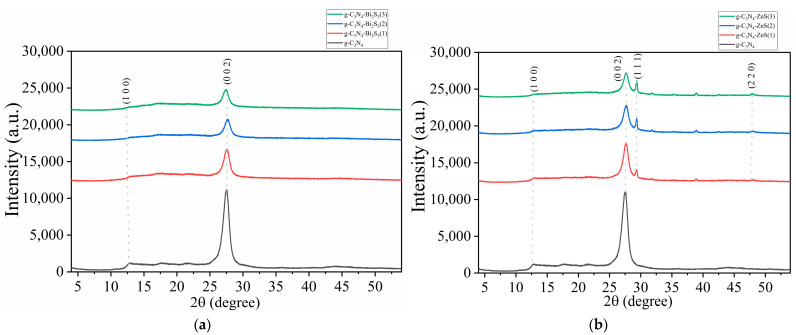
XRD spectra of (**a**) g-C_3_N_4_ and g-C_3_N_4_-Bi_2_S_3_; (**b**) g-C_3_N_4_, and g-C_3_N_4_-ZnS.

The X-ray diffraction (XRD) pattern of g-C_3_N_4_, g-C_3_N_4_-Bi_2_S_3_, and g-C_3_N_4_-ZnS composites (Figure 2a,b) elucidates the structural characteristics of graphitic carbon nitride (g-C_3_N_4_), bismuth sulfide (Bi_2_S_3_), and zinc sulfide (ZnS) phases. Pure g-C_3_N_4_ (black curve) has a significant peak at around 27.4°, corresponding to the (002) plane, indicating the layered stacking architecture of the g-C_3_N_4_ sheets. This peak is indicative of g-C_3_N_4_ and signifies its layered structure, similar to that of graphite. A supplementary, diminished peak often appears at 13.1°, correlating with the (100) plane and signifying the in-plane structural arrangement of the heptazine units constituting g-C_3_N_4_. Collectively [27], these peaks confirm the layered and crystalline structure of g-C_3_N_4_.

In Figure 2a, the peaks maintain their position and shape despite the introduction of Bi_2_S_3_ at different concentrations: g-C_3_N_4_-Bi_2_S_3_(1), g-C_3_N_4_-Bi_2_S_3_(2), and g-C_3_N_4_-Bi_2_S_3_(3) are shown by red, blue, and green curves, respectively. The absence of shift or widening in the primary peaks suggests that Bi_2_S_3_ does not substantially modify the crystalline structure of g-C_3_N_4_.

The figure also indicates a decrease in peak intensity with increasing Bi_2_S_3_ concentration. This indicates that increased quantities of Bi_2_S_3_ in the composite may obscure the diffraction signals of g-C_3_N_4_, diminishing the overall strength of the g-C_3_N_4_ peaks at about 13.1° and 27.4°. Notwithstanding the reduction in intensity, the locations and configurations of these peaks remain stable, indicating that the g-C_3_N_4_ structure is not substantially affected by the incorporation of Bi_2_S_3_ [28]. The result is consistent with the literature, which indicates that Bi_2_S_3_ does not significantly compromise the crystalline structure of g-C_3_N_4_.

In Figure 2b, upon the incorporation of ZnS, supplementary peaks appear at 28.5° and 47.5°, corresponding to the (111) and (220) planes of ZnS, respectively. The observed peaks correspond to the zinc blende (cubic) phase of ZnS, indicating that ZnS crystallizes in this structure within the composite [29]. The intensity and sharpness of these ZnS peaks indicate a high crystalline degree of ZnS in the composite, with no structural deformation. The simultaneous existence of g-C_3_N_4_ and ZnS peaks, without widening, suggests that the components are uniformly disseminated and that ZnS nanoparticles maintain their crystallinity throughout composite synthesis.

Figure 2b shows augmentation of ZnS concentration in the composite, as seen by samples g-C_3_N_4_-ZnS(1), g-C_3_N_4_-ZnS(2), and g-C_3_N_4_-ZnS(3) depicted by red, blue, and green curves, respectively, leading to an increased intensity of the ZnS peaks. This indicates that an increased amount of ZnS enhances the crystalline and volume fraction of ZnS inside the composite, which is anticipated to improve the photocatalytic and electronic characteristics of the composite owing to enhanced interfacial contact between g-C_3_N_4_ and ZnS. The use of starch as a modifying agent facilitated the uniform dispersion of ZnS particles, thus enhancing the composite’s structural stability and interfacial contact, hence strengthening its functional properties.

The green curve in Figure 2a,b, indicating the highest Bi_2_S_3_ and ZnS concentration, exhibits a little leftward shift in the principal g-C_3_N_4_ peaks at about 13.1° and 27.4° (2θ). This alteration indicates that increased Bi_2_S_3_ and ZnS concentrations may induce small interactions or strain at the g-C_3_N_4_-Bi_2_S_3_ and g-C_3_N_4_-ZnS interfaces. This change may signify minor lattice deformation in the g-C_3_N_4_ structure, either resulting from the integration of Bi_2_S_3_ and ZnS nanoparticles or the interfacial tension arising from composite production. The leftward shift indicates an augmentation in the d-spacing between layers, possibly resulting from Bi_2_S_3_ and ZnS nanoparticles influencing the stacking configuration of g-C_3_N_4_. While Bi_2_S_3_ and ZnS do not change the underlying crystalline structure of g-C_3_N_4_, the increased amount seems to significantly influence the interlayer gap. This little alteration, along with diminished intensity, elucidates how increased Bi_2_S_3_ and ZnS concentrations may cautiously influence the XRD spectra of g-C_3_N_4_ without completely compromising its structure.

### 2.3. SEM Analysis

**Figure 3:**
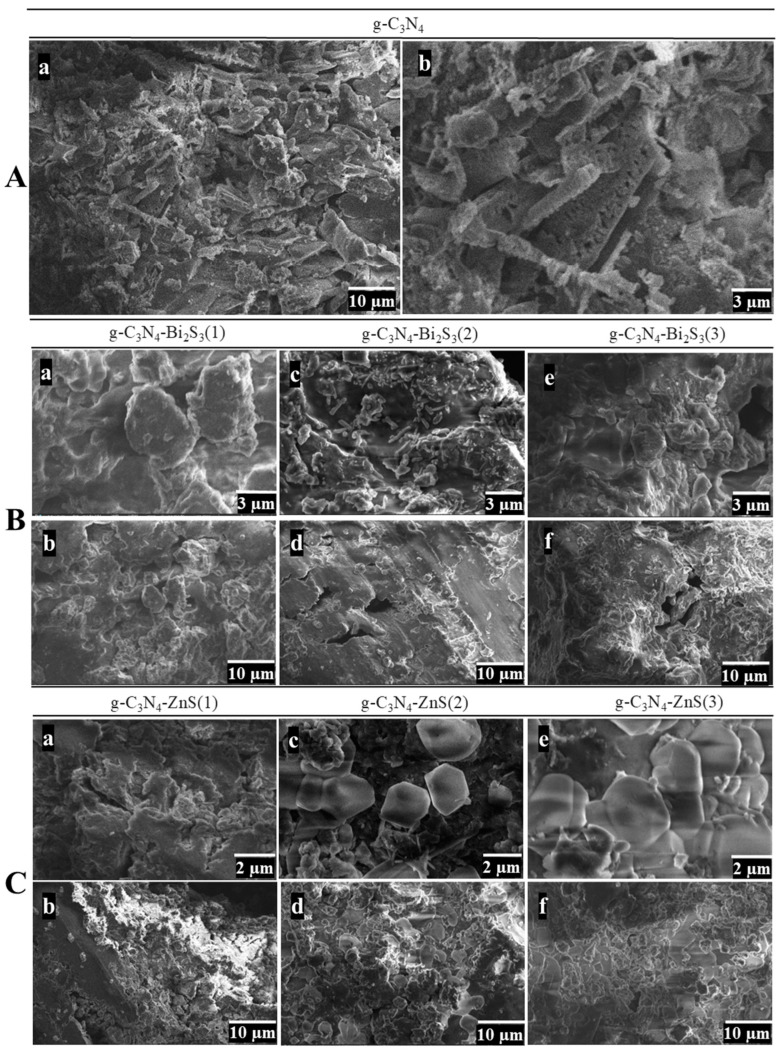
SEM images of (**A**) g-C_3_N_4_ at low (5000×) (**a**) and high (15,000×) (**b**) magnification. (**B**) g-C_3_N_4_-Bi_2_S_3_ at high (15,000×) (**a**,**c**,**e**) and low (5000×) (**b**,**d**,**f**) magnification. (**C**) g-C_3_N_4_-ZnS at high (20,000×) (**a**,**c**,**e**) and low (5000×) (**b**,**d**,**f**) magnification.

The SEM morphology of g-C_3_N_4_ exhibits a multi-layered, porous structure that optimizes the surface area and the accessibility of active sites, which is crucial for its role as a photocatalyst, as shown in Figure 3A(a,b).

The low-magnification image (Figure 3A(a)) reveals the characteristic layered, flake-like morphology of g-C_3_N_4_. This morphology is indicative of graphitic carbon nitride, which inherently develops stacked sheets owing to the layered configuration of its atomic structure [30]. The picture depicts loosely arranged sheets with jagged edges and an uneven configuration. These characteristics provide a comparatively large surface area, advantageous for photocatalytic applications since it offers sufficient room for reactant adsorption.

At high magnification (Figure 3A(b)), the intricacies of these layers become more apparent. The individual sheets are more clearly defined, exhibiting visible gaps and cracks between them. This emphasizes the stratified, permeable structure. The edges of the sheets seem tattered and irregular, perhaps increasing catalytic activity by revealing more active sites. This course, permeable structure is beneficial for photocatalysis since it enhances light absorption and interaction with contaminants, possibly increasing degradation efficiency.

The SEM images of the g-C_3_N_4_-Bi_2_S_3_ composites in Figure 3B(a–f) illustrate how varying concentrations of Bi_2_S_3_ affect the surface morphology and structural characteristics of the material. In the sample with the lowest Bi_2_S_3_ concentration, g-C_3_N_4_-Bi_2_S_3_(1), the high-magnification image (Figure 3B(a)) reveals an aggregated, granular texture with rounded particles lacking distinctive shapes. At lower magnification (Figure 3B(b)), the morphology appears relatively uniform, showing larger aggregates likely formed by the g-C_3_N_4_ matrix. This uniform, featureless appearance suggests that the limited Bi_2_S_3_ concentration does not significantly alter the structure of g-C_3_N_4_, resulting in a dispersed or weakly clustered deposition that does not create defined morphological features.

A marked change occurs in the g-C_3_N_4_-Bi_2_S_3_(2) sample, where rod-like structures are evident in the high-magnification image (Figure 3B(c)) [31]. These rod formations indicate that the intermediate concentration of Bi_2_S_3_ promotes anisotropic growth, with Bi_2_S_3_ nanocrystals forming elongated shapes on the g-C_3_N_4_ surface. This rod-like structure is likely to enhance photocatalytic efficiency by creating additional surface area and reaction sites. At a lower magnification (Figure 3B(d)), these rod-like structures are seen more extensively across the sample, embedded in the g-C_3_N_4_ matrix, highlighting an improved interfacial interaction between Bi_2_S_3_ and g-C_3_N_4_ at this concentration.

However, in the g-C_3_N_4_-Bi_2_S_3_(3) sample, the morphology changes again, as seen at high magnification (Figure 3B(e)), where densely packed clusters replace the distinct rod-like formations observed in the previous sample. This agglomeration suggests that the higher Bi_2_S_3_ concentration leads to excessive coverage on the g-C_3_N_4_ surface, potentially reducing the accessible surface area and hindering photocatalytic performance due to blocked active sites. At lower magnification (Figure 3B(f)), the material appears compact and rough, with few distinguishable features, indicating that the higher Bi_2_S_3_ concentration oversaturates the g-C_3_N_4_ surface, leading to a loss of morphological detail and, likely, reduced effectiveness in pollutant degradation.

The SEM images of g-C_3_N_4_-ZnS composites in Figure 3C(a–f) illustrate the impact of varying ZnS concentrations on the material’s morphology, specifically for g-C_3_N_4_-ZnS(1), g-C_3_N_4_-ZnS(2), and g-C_3_N_4_-ZnS(3), which correspond to additional volumes of 25 cm^3^, 45 cm^3^, and 65 cm^3^ of 10 mM ZnS, respectively. In the sample with the lowest ZnS content, g-C_3_N_4_-ZnS(1), the high-magnification picture (Figure 3C(a)) displays an irregular, coarse surface morphology characterized by tiny, aggregated particles distributed throughout the g-C_3_N_4_ matrix. The low-magnification image (Figure 3C(b)) further depicts a coarse and comparatively porous structure, characterized by discernible voids within particle clusters, signifying a reduced density of ZnS on the g-C_3_N_4_ surface.

In g-C_3_N_4_-ZnS(2), with an increased ZnS concentration, the high-magnification picture (Figure 3C(c)) reveals the formation of a bigger and hexagonal morphology, with more defined clusters of ZnS particles throughout the g-C_3_N_4_ matrix [31]. These clusters seem to encompass the surface more comprehensively than in g-C_3_N_4_-ZnS(1), indicating enhanced distribution and interaction between ZnS and g-C_3_N_4_. At low magnification (Figure 3C(d)), the overall structure retains its porosity, while the ZnS clusters exhibit a more uniform distribution, potentially augmenting the material’s surface area and the active sites accessible for photocatalytic processes. The increased concentration of ZnS results in a more uniform coating on the g-C_3_N_4_ substrate.

In g-C_3_N_4_-ZnS(3) with the greatest ZnS concentration, the high-magnification image (Figure 3C(e)) reveals densely aggregated ZnS clusters that create extensive, plate-like formations, encompassing substantial areas of the g-C_3_N_4_ surface. The substantial ZnS covering signifies saturation, perhaps resulting in a reduced exposed surface area of g-C_3_N_4_. The low-magnification image (Figure 3C(f)) reveals a compact, less porous structure in contrast to the preceding samples, characterized by a dense aggregation of ZnS particles. This extensive covering may impede photocatalytic activity by diminishing light penetration and obstructing active areas on the g-C_3_N_4_ surface, thereby restricting pollutant interaction. The images demonstrate a transition from scattered to more continuous ZnS layers as the concentration rises, with ideal dispersion seen at intermediate concentrations.

### 2.4. TEM Analysis

**Figure 4:**
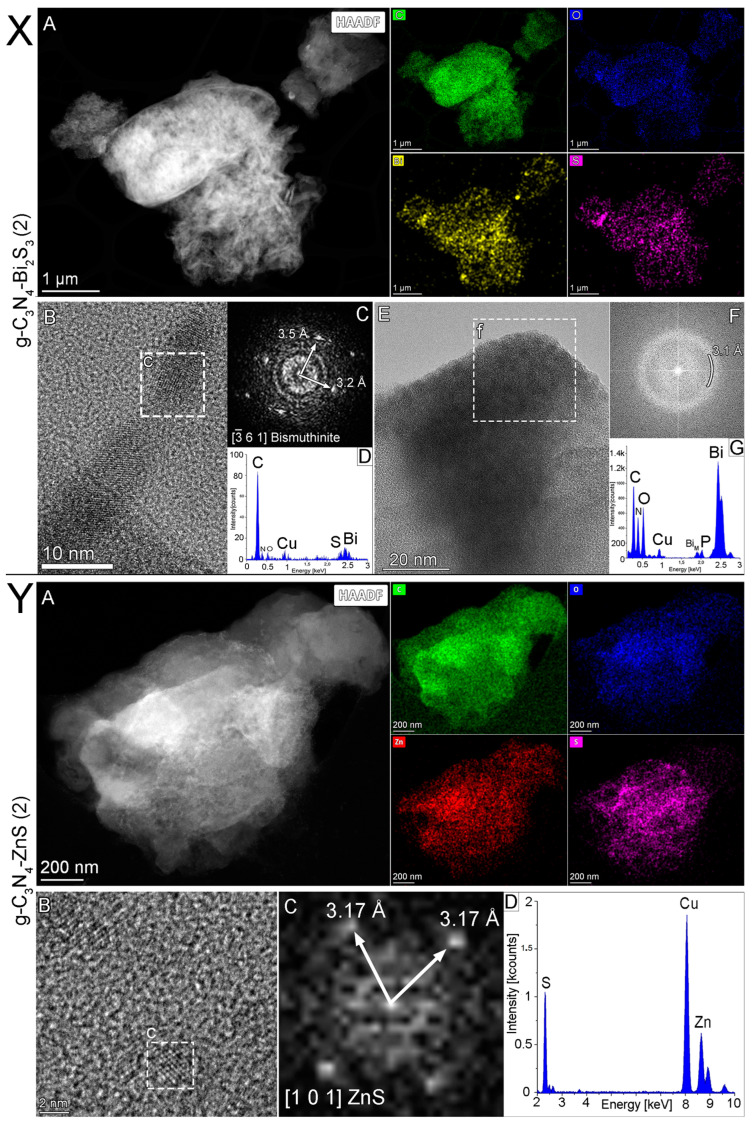
(**X**) Scanning transmission electron microscopy analysis of g-C_3_N_4_-Bi_2_S_3_ (2), (**A**) STEM-HAADF image and STEM-EDS element maps showing the distributions of carbon (green), oxygen (blue), sulfur (pink), and bismuth (yellow), (**B**) HRTEM image shows crystalline structure (c) of the nanoparticles on the carrier, (**C**) fast Fourier transform image of the (c) marked particle confirming the structure to be of bismuthinite (Bi_2_S_3_), aligned with the electron beam parallel to the [−3 6 1] crystallographic direction, and (**D**) EDS spectrum of particle (c), obtained from the STEM-EDS elemental map. (**E**) HRTEM image shows the amorphous nature (f) of the nanoparticles on the carrier, (**F**) fast Fourier transform image of the (f) marked area, confirming the structure to be amorphous, it can be characterized with a diffraction ring of 3.1 Å radius. (**G**) EDS spectrum of the (f) marked area shows that the particle contains mainly bismuth with high amounts of oxygen (and C), suggesting the particles to be likely bismuth oxide or bismuthite. (**Y**) Scanning transmission electron microscopy analysis of g-C_3_N_4_-ZnS(2), (**A**) STEM-HAADF image and STEM-EDS element maps showing the distributions of carbon (green), oxygen (blue), sulfur (pink), and zinc (red), (**B**) HRTEM image shows crystalline nanoparticles (c) on the carbon nitride carrier, and (**C**) fast Fourier transform image of the (c) marked particle confirming the structure to be of sphalerite (ZnS), aligned with the electron beam parallel to the [101] crystallographic direction, and (**D**) The EDS spectrum of the area (**B**), obtained from the STEM-EDS elemental map.

TEM analysis was conducted on the selected optimal nanocomposite, namely the g-C_3_N_4_-Bi_2_S_3_(2) and g-C_3_N_4_-ZnS(2). Figure 4X displays a low-magnification scanning transmission electron microscopy examination of Bi_2_S_3_-modified graphitic carbon nitride. Both the STEM-HAADF picture and the STEM-EDS element maps illustrate the distributions of carbon (in green), oxygen (in blue), sulfur (in pink), and bismuth (in yellow). The sample has a rather uniform distribution of bismuth and sulfur; however, the bigger particles that contain bismuth have a strong O signal with a low S signal, which indicates the existence of bismuth oxide in addition to bismuth sulfide. Additionally, the elemental maps are supported by the EDS spectrum of the whole HAADF picture (Figure 4X(D)).

Transmission electron microscopy examination of Bi_2_S_3_ graphitic carbon nitride was conducted, and the HRTEM picture (Figure 4X(E)) appears to show an amorphous character of the nanoparticle (f); also, a fast Fourier transform of the highlighted region (Figure 4X(E/f)) shows only a diffraction ring with a radius of 3.1 Å. The EDS spectrum (Figure 4X(G)) of the region (Figure 4X(E/f)) indicates that the particle mostly comprises bismuth, with significant quantities of oxygen (and carbon), suggesting that the particles are likely bismuth oxide. The presence of copper in EDS spectra is due to the copper grid used for the TEM analysis [32]. Notably, control experiments were carried out with an Au grid, and no copper was detected.

The HRTEM image (Figure 4X(B)) displays the crystalline structure (Figure 4X(B/c)) of Bi_2_S_3_ nanoparticles on the g-C_3_N_4_ carrier. The EDS spectrum (Figure 4X(D)) and the fast Fourier transform (Figure 4X(C)) image of the marked particle (Figure 4X(B/c)) corroborate that the structure is bismuthinite (Bi_2_S_3_), with the electron beam oriented parallel to the [−3 6 1] crystallographic direction. Bismutite (Bi_2_(CO_3_)O_2_) manifests as an oxidation product of various bismuth minerals, including bismuthinite (Bi_2_S_3_) and native bismuth. The EDS spectrum in Figure 4X(D) also suggests elevated carbon levels compared to carrier graphitic carbon nitride, suggesting its potential presence as well. The bismuth to oxygen ratio is 10:18, which is more akin to Bi_2_O_3_.

Figure 4Y illustrates the scanning transmission electron microscopy study of the best ZnS-modified graphitic carbon nitride. The STEM-HAADF picture Figure 4Y(A) and the STEM-EDS elemental maps (Figure 4Y(D)) illustrate the distributions of carbon (green), nitrogen (blue), sulfur (pink), and zinc (yellow). The EDS spectrum of the whole HAADF picture indicates that the sample mostly comprises carbon and nitrogen, with significant quantities of zinc and sulfur [32]. The presence of copper in EDS spectra is due to the copper grid used for the TEM analysis.

The crystalline zinc sulfide nanoparticles are shown in the HRTEM picture (Figure 4Y(B)) in Figure 4, whereas the region indicated with (c) demonstrates a 2 nm-large zinc sulfide nanoparticle on the carbon-nitride carrier. The evidence that the structure is of sphalerite (ZnS) is provided by the fast Fourier transform (C) of the nanoparticle that has been designated (Figure 4Y(B/c)). The lattice fringe spacing of 3.17 Å, as shown in the image, corresponds to the two (111)-type (such as (111) and (1-1-1)) crystallographic planes of ZnS, visible in this [101] orientation. This confirms the presence of crystalline ZnS nanoparticles within the composite structure [33].

### 2.5. BET Surface Analysis

The BET surface area is crucial in assessing the photocatalytic efficacy of materials such as g-C_3_N_4_ and its composites, since it directly influences the availability of active sites for light absorption and pollutant decay. The BET surface area of pristine g-C_3_N_4_ is notably high at 27.9 m^2^/g, providing an increased quantity of accessible active sites for photocatalysis. Nonetheless, the formation of composites using Bi_2_S_3_ and ZnS that utilize starch as a template or modifying agent resulted in a substantial reduction in the BET surface area for both composites, as seen in Table S2. This decrease may be attributed to several processes, such as surface covering, structural densification, and the combined effect of later phases.

The reduction in BET surface area observed in the starch-assisted synthesis of g-C_3_N_4_- Bi_2_S_3_ and g-C_3_N_4_-ZnS composites is likely attributable to the deposition of Bi_2_S_3_ or ZnS onto the g-C_3_N_4_ framework, which may occlude or obstruct its inherent pores, hence restricting surface accessibility during BET analysis. Starch, throughout the entire producing process, might break down, leaving behind residues or changing the composite structure. This may result in agglomeration or densification, hence decreasing porosity and the accessible surface area. The leftover organic matter from starch degradation may also occupy surface sites, thus further diminishing the surface area.

Although the BET surface area decreases, the integration of starch in the fabrication of g-C_3_N_4_- Bi_2_S_3_ and g-C_3_N_4_-ZnS composites produces synergistic effects that improve photocatalytic efficacy. Starch facilitated the formation of heterojunctions, enhancing charge carrier separation by promoting the transport of photogenerated electrons and holes between the materials, hence diminishing recombination. This effective charge separation improves the production of reactive species such as ^•^OH and O_2_^•−^, which are crucial for pollutant degradation. Furthermore, the composites broadened the light absorption spectrum owing to their low band gaps, facilitating the enhanced usage of visible light. These variables mitigate the decreased surface area by enhancing overall light absorption and catalytic efficacy.

Overall, the BET surface area decreased from 22 m^2^/g for g-C_3_N_4_ to 12.9, 6.9, and 8.9 m^2^/g for g-C_3_N_4_-Bi_2_S_3_(1), g-C_3_N_4_-Bi_2_S_3_(2), and g-C_3_N_4_-Bi_2_S_3_(3), as well as 4.2, 2.6, and 2.6 m^2^/g for g-C_3_N_4_-ZnS(1), g-C_3_N_4_-ZnS(2), and g-C_3_N_4_-ZnS(3) composites synthesized with starch—this reduction signifies a decrease in active surface sites. However, it concurrently facilitates improved charge separation, expanded light absorption, and synergistic interactions within the composites, which markedly enhance photocatalytic efficacy. The balance between surface area and enhanced functionality highlights the essential function of starch in the design of an effective photocatalytic system, where heterojunction formation and functionalization are pivotal for attaining high catalytic efficiency.

### 2.6. XPS Analysis

XPS investigation can demonstrate synergistic effects between g-C_3_N_4_ and Bi_2_S_3_, as well as between g-C_3_N_4_ and ZnS in the nanocomposite. XPS is a useful method for assessing the surface composition of the nanocomposites, chemical states, and possible interactions between the components.

**Figure 5:**
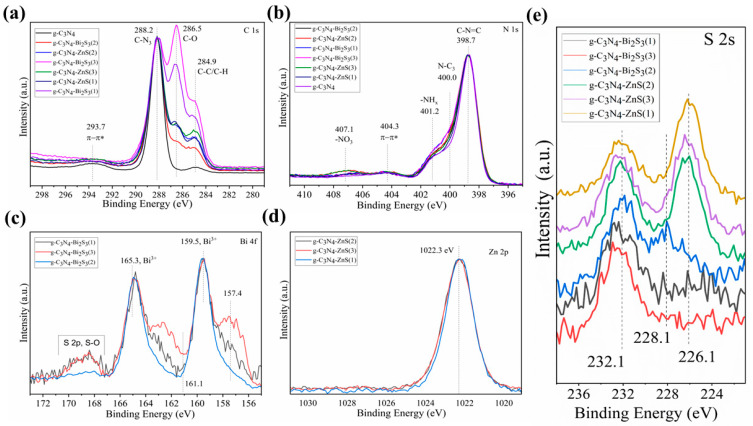
Comprehensive XPS spectra of g-C_3_N_4_-Bi_2_S_3_ and g-C_3_N_4_-ZnS nanocomposites. XPS spectra of (**a**) C 1s (**b**) N 1s (**c**) Bi 4f (**d**) Zn 2p and (**e**) S 2s.

The results of an XPS investigation of the valence state and chemical environment of the component elements in the g-C_3_N_4_, g-C_3_N_4_-Bi_2_S_3_, and g-C_3_N_4_-ZnS nanocomposites are shown in Figure 5. The distinctive review spectrum (Figure 5a–e) indicates the presence of C 1s, N 1s, O 1s, Bi 4f, Zn 2p, and S 2s elements. The lack of other peaks indicates that the nanocomposite g-C_3_N_4_-Bi_2_S_3_ and g-C_3_N_4_-ZnS primarily include the elements C, N, O, S, Bi, and Zn.

The C 1s spectrum in Figure 5a clearly indicates that carbon exists in several chemical surroundings. The black curve representing g-C_3_N_4_ has a small C-C/C-H component due to the adventitious carbon. The high-resolution spectrum of C 1s also displays three prominent peaks at binding energy values of 288.2, 286.5, and 284.9 eV, which correspond to graphite C-N_3_ within the aromatic g-C_3_N_4_ lattice, as well as C-O and C-C/C-H peaks resulting from interactions with the functional groups of starch, utilized as a stabilizing and reducing agent, respectively [34].

Three major peaks in pyridine-like aromatic structures are seen in the high-resolution N 1 s spectra (Figure 5b). These peaks are located at 398.7, 400.0, and 401.2 eV and may be ascribed to C-N=C, tertiary nitrogen N-(C)_3_, and -NH_2_ [35]. The metal nitrate salt used in the synthesis is responsible for the peak at 407.1 eV.

The π bond shake-up satellites at 293.7 in the C 1s spectrum and at 404.3 eV in the N 1s spectrum are characteristic of the graphitic carbon nitride [36].

The chemical states of Bi and S in the Bi_2_S_3_ nanoparticles can be observed in the high-resolution Bi 4f spectra (Figure 5c). The binding energies for Bi 4f_7/2_ and Bi 4f_5/2_ are seen in the (Bi 4f) spectra at 159.5 eV and 165.3 eV, respectively [37]. The S 2p_3/2_ photoelectron peak of a sulfide in Bi_2_S_3_ should appear at around 161.1 eV; however, it overlaps with the Bi 4f dublet. Only the S 2p dublet of the S-O bond—due to the several eV chemical shifts—is visible in the Bi 4f region without obstruction [38]. Because of the overlapping regions, the S 2s spectrum (Figure 5e) was assessed to demonstrate the existence of sulfur.

The Zn 2p core-level XPS spectra (Figure 5d) exhibited a distinctive peak at 1022 eV.3 eV, which is attributed to the Zn 2p_3/2_ areas, providing clear evidence of the presence of Zn 2p [39].

The high-resolution XPS peak of S 2s spectra (Figure 5e) suggests that the three zinc samples (upper spectra) exhibiting peaks at 226.1 and 232.1 eV may correspond to zinc sulfide and oxygen-bound sulfur due to the presence of starch. The three lower spectra correspond to bismuth sulfide samples, as demonstrated by the peak at 228.1 eV.

Due to the mass balance, it is quite clear that there are not only Bi_2_S_3_ and ZnS on the surface but also that oxide is quite possible, as Bi_2_O_3_ and Bi_2_S_3_ are quite close to each other in binding energy, as are ZnO and ZnS. These results are in accordance with those of the ICP measurements.

### 2.7. Comparison of Atomic Weight % by XPS and EDX Analysis

For all of the synthesized composites, XPS has been compared with EDX in the SM, Table S3.

The comparison of EDX (Energy-Dispersive X-ray Spectroscopy) and XPS (X-ray Photoelectron Spectroscopy) results for g-C_3_N_4_, g-C_3_N_4_-Bi_2_S_3_, and g-C_3_N_4_-ZnS indicates significant disparities in the identified elemental composition. The inconsistencies come from the fundamental differences in the methodologies. EDX offers insights into bulk elemental composition with less surface sensitivity, whereas XPS is a surface-sensitive method that examines the upper few nanometers of the material.

For g-C_3_N_4_: EDX indicates a greater carbon content (47.39%) than XPS (41.45%). Nitrogen is found to be more prevalent in XPS (57.76%) compared to EDX (43.9%). This indicates a higher nitrogen content at the surface relative to the bulk. The oxygen content identified by EDX has increased (8.71%), which is presumably traceable to adsorbed oxygen species in the bulk or absorbing environment.

For g-C_3_N_4_-Bi_2_S_3_: In all g-C_3_N_4_-Bi_2_S_3_ samples (1, 2, and 3), XPS consistently reveals increased amounts of sulfur and bismuth relative to EDX, suggesting that Bi_2_S_3_ is primarily distributed on the surface. For example, in g-C_3_N_4_-Bi_2_S_3_(3), sulfur constitutes 0.27% in XPS and 0.11% in EDX; however, bismuth is much more in XPS at 0.1% compared to 0.06% in EDX. This tendency is consistent across all ratios, indicating that the Bi_2_S_3_ phase is surface dominated in these composites.

For g-C_3_N_4_-ZnS: In the g-C_3_N_4_-ZnS samples (1, 2, and 3), XPS indicates enhanced sulfur and zinc concentrations relative to EDX, signifying ZnS enrichment at the surface. For instance, in g-C_3_N_4_-ZnS(1), XPS indicates a zinc concentration of 1.75%, but EDX reveals a concentration of 0.16%. Additionally, sulfur content is greater in XPS at 0.73% compared to 0.11% in EDX. The trend is uniform across all ratios, suggesting that ZnS mostly adorns the surface.

The elemental analysis illustrates the complementary characteristics of EDX and XPS. The surface concentration of Bi_2_S_3_ and ZnS indicates efficient dispersion and persistent surface interactions—essential for improving photocatalytic activities. The detected disparities between the two methodologies emphasize the significance of surface analysis in the characterization of heterostructure materials.

### 2.8. UV–Vis Diffuse Reflectance Analysis

UV–Vis DRS analysis was used to analyze the optical characteristics of the as-synthesized pure g-C_3_N_4_, g-C_3_N_4_-Bi_2_S_3_(1), g-C_3_N_4_-Bi_2_S_3_(2), g-C_3_N_4_-Bi_2_S_3_(3), g-C_3_N_4_-ZnS(1), g-C_3_N_4_-ZnS(2), and g-C_3_N_4_-ZnS(3) (Figure S1). Both g-C_3_N_4_-Bi_2_S_3_ and g-C_3_N_4_-ZnS exhibited considerable light absorption in a broad visible spectrum. The nanocomposite g-C_3_N_4_-Bi_2_S_3_ and g-C_3_N_4_-ZnS demonstrated a moderately enhanced absorption range in the visible light spectrum. The band gap energies of pure g-C_3_N_4_, g-C_3_N_4_-Bi_2_S_3_ and g-C_3_N_4_-ZnS nanocomposites were measured using the Tauc relation, as seen in the following equation (Equation (1)) [40].

Ahν = A(hν − Eg)^n/2^ (1)

The proportionality constant, absorption coefficient, light frequency, band gap energy, and Planck constant are represented by the letters A, α, v, Eg, and h, respectively.

According to the Tauc plot (Figure S2), the band gap energies of the nanocomposite have been determined and presented in Table S4. The inaccuracy of the band gap is around 0.01–0.02 eV, hence the resultant differences are not significant. A reduction in BG value may lead to an increase in absorbed photons and thus enhanced catalytic efficiency; nevertheless, a drop of 0.1 eV yields only a 5–10% increase in absorbed photons. The band-gap energy of Bi_2_S_3_ nanocomposite does not significantly influence photoactivity; however, in the case of ZnS nanocomposite, the band-gap energy decreases further into the visible spectrum, predicting an efficient photoactivity for coumarin and para-nitrophenol.

### 2.9. Photoactivity Results

The photocatalytic process initiated by the irradiation of a photoactive semiconductor, such as g-C_3_N_4_, produces photo-generated electrons in the conduction band and positive holes in the valence band (Equation (2)). The electrons in the conduction band can react with O_2_, generating superoxide radicals (Equation (3)). The holes in the valence band can undergo reaction with water to produce hydrogen ions and hydroxyl radicals (Equation (4)). A similar reaction takes place with hydroxide ions, generating ^•^OH (Equation (5)). Superoxide and hydroxyl radicals are important in the degradation of organic contaminants.

g-C_3_N_4_ + hν → e^−^_CB_ + h^+^_VB_ (2)

e^−^_CB_ + O_2_ → O_2_^•−^ (3)

h^+^_VB_ + H_2_O → ^•^OH + H^+^ (4)

h^+^_VB_ + OH^−^ → ^•^OH (5)

These are the primary possible reactions after the excitation of photoactive semiconductors in air-saturated aqueous systems. The reactive species formed can undergo further reactions with each other, producing additional oxidizing compounds such as H_2_O_2_.

#### 2.9.1. Photoactivity Results of g-C_3_N_4_-Bi_2_S_3_

The photodegradation of coumarin and para-nitrophenol was evaluated utilizing various fabricated bismuth sulfide photocatalysts by exposing an aqueous solution of the colorless organic pollutants to UV and visible light, accompanied by continuous air bubbling. Before being exposed to light, the pollutant-contaminated water containing the organic pollutant and photocatalyst suspension was left in the dark for about half an hour while being constantly stirred to ensure that adsorption and desorption were balanced.

**Figure 6:**
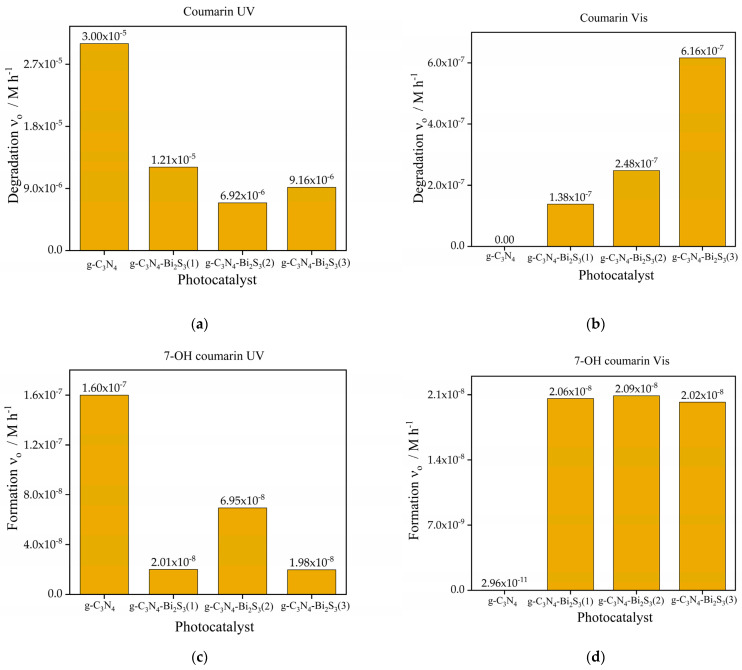
Initial rate of degradation of coumarin (**a**,**b**) and formation 7-OH coumarin (**c**,**d**) under UV (**a**,**c**) and visible (**b**,**d**) light with g-C_3_N_4_ and g-C_3_N_4_-Bi_2_S_3_ (with 3 different compositions).

The initial rate of coumarin degradation under UV light is higher for pure g-C_3_N_4_, as seen in Figure 6a, demonstrating its high efficiency in photocatalytic UV exposure. Nonetheless, when the concentration of Bi_2_S_3_ increases in the modification of g-C_3_N_4_, the degradation rate progressively diminishes. An example is g-C_3_N_4_-Bi_2_S_3_(1), which has a reduced degradation rate relative to pure g-C_3_N_4_ (1.21 × 10^−^^5^ M h^−1^), with additional reductions seen in g-C_3_N_4_-Bi_2_S_3_(2) and g-C_3_N_4_-Bi_2_S_3_(3). This indicates that increased Bi_2_S_3_ concentrations inhibit the catalytic efficacy of g-C_3_N_4_ for coumarin under UV illumination, perhaps because Bi_2_S_3_ is less efficient at absorbing and utilizing UV photons than g-C_3_N_4_.

Under UV illumination, the initial rate of 7-OH coumarin production (Figure 6c) shows a similar pattern. Indicating considerable hydroxyl radical (^•^OH) generation during photocatalysis, pure g-C_3_N_4_ produces the greatest rate of 7-OH coumarin formation (1.6 × 10^−^^7^ M h^−1^). By contrast, with the rate of production decreasing as the Bi_2_S_3_ concentration rises, Bi_2_S_3_-modified catalysts generate less ^•^OH radicals. The lowest production rate of 7-OH coumarin for g-C_3_N_4_-Bi_2_S_3_(3) (1.98 × 10^−^^8^ M h^−1^) implies that adding Bi_2_S_3_ lowers the creation of ^•^OH radicals under UV radiation. Bi_2_S_3_ could overshadow g-C_3_N_4_ UV-driven photocatalytic activity, so this reduction might be ascribed to a diminished capacity of the composite to enable the reactions producing ^•^OH radicals.

The initial rate of coumarin degradation under visible light, as shown in Figure 6b, is minimal for pure g-C_3_N_4_, demonstrating its ineffectiveness in harnessing visible light owing to its comparatively broad bandgap. The incorporation of Bi_2_S_3_ markedly improves visible light activity due to its reduced band gap, enabling it to function as a photosensitizer. The degradation rate escalates with increasing Bi_2_S_3_ concentration, with g-C_3_N_4_-Bi_2_S_3_(3) demonstrating the highest (6.16 × 10^−^^7^ M h^−1^) initial degradation rate. This trend underscores the pivotal role played by Bi_2_S_3_ in enhancing visible-light-driven photocatalysis by broadening light absorption into the visible spectrum and promoting charge separation inside the composite. The initial rate of 7-OH coumarin production under visible light, as shown in Figure 6d, is minimal for pure g-C_3_N_4_ (2.96 × 10^−^^11^ M h^−1^) but becomes substantial with Bi_2_S_3_ alteration. All three Bi_2_S_3_-modified catalysts exhibit similar rates of 7-OH coumarin formation, suggesting that the generation of hydroxyl radicals (^•^OH) is mostly facilitated by Bi_2_S_3_ under visible light. Notably, the formation of ^•^OH radicals does not seem to be significantly influenced by the concentration of Bi_2_S_3_, indicating that even at low concentrations, Bi_2_S_3_ efficiently promotes visible light absorption and the ensuing reactions that yield ^•^OH radicals. On the contrary, the degradation rate of coumarin steeply increased with higher concentrations of Bi_2_S_3_. This phenomenon suggests that hydroxyl radicals are not the exclusive oxidizing species in this case. For instance, direct reaction of coumarin with the photogenerated holes cannot be excluded either. This is promoted by trapping the electrons in the conduction band of Bi_2_S_3_, the potential of which (0.12 V [41]) is far below that of g-C_3_N_4_ (−1.16 V [42]). This low potential, however, can diminish the production of superoxide radical anions (O_2_^•−^) via the reaction between dissolved oxygen and e^−^_CB_ because its redox potential is −0.12 V [43].

**Figure 7:**
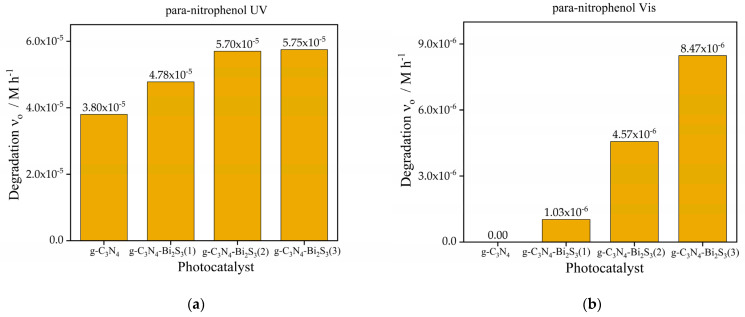
Initial rate of degradation of para-nitrophenol under (**a**) UV and (**b**) visible light.

The initial rate of para-nitrophenol degradation, as shown in Figure 7a is markedly increased by the incorporation of Bi_2_S_3_ into g-C_3_N_4_ under UV light. Although pure g-C_3_N_4_ exhibits low degradation activity (3.8 × 10^−^^5^ M h^−1^), all Bi_2_S_3_-modified composites surpass it, emphasizing the synergistic benefits of integrating g-C_3_N_4_ with Bi_2_S_3_ under UV illumination. The modified catalysts g-C_3_N_4_-Bi_2_S_3_(2) and g-C_3_N_4_-Bi_2_S_3_(3) demonstrate comparable and somewhat increased degradation rates relative to g-C_3_N_4_-Bi_2_S_3_(1). This signifies that at moderate to higher Bi_2_S_3_ concentrations, the composite attains an ideal optimum of UV light absorption and charge separation efficiency, resulting in enhanced photocatalytic activity. The improved efficacy of the Bi_2_S_3_-modified catalysts under UV illumination indicates that Bi_2_S_3_ facilitates superior charge separation by functioning as an electron acceptor. In contrast to coumarin degradation (Figure 6a), the incorporation of Bi_2_S_3_ in these composites did not inhibit UV-induced activity for para-nitrophenol degradation, maybe owing to distinct reaction pathways or active species engaged in the degradation process.

Initially, para-nitrophenol degradation is insignificant for pure g-C_3_N_4_ under visible-light irradiation (Figure 7b) because its absorption in the visible range is very limited, and the recombination of the charge carriers is considerably efficient. However, adding Bi_2_S_3_ significantly boosts activity, with degradation rates rising with higher concentrations. The maximum degradation rate is shown with g-C_3_N_4_-Bi_2_S_3_(3) (8.47 × 10^−^^6^ M h^−1^), followed by g-C_3_N_4_-Bi_2_S_3_(2) (4.57 × 10^−^^6^ M h^−1^) and g-C_3_N_4_-Bi_2_S_3_(1) (1.03 × 10^−^^6^ M h^−1^). Increasing Bi_2_S_3_ concentration coincides with this trend, indicating its importance as a visible-light photosensitizer. Bi_2_S_3_-modified catalysts absorb visible light photons and promote the production of reactive oxidizing species for para-nitrophenol degradation, resulting in increased visible light activity. The g-C_3_N_4_-Bi_2_S_3_ interface efficiently separates photogenerated electron hole pairs, preventing recombination (through trapping charge carriers) and increasing photocatalytic activity in visible light.

**Figure 8:**
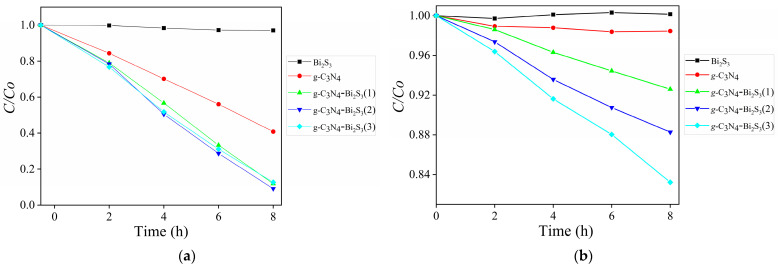
C/C_0_ curves for para-nitrophenol degradation by irradiations with g-C_3_N_4_-Bi_2_S_3_ (**a**) UV and (**b**) visible LEDs.

Figure 8 illustrates the degradation efficiency of para-nitrophenol (C/C_0_ vs time) under UV (a) and visible light (b) using various photocatalysts. Under UV illumination, pure g-C_3_N_4_ exhibits moderate activity owing to its robust absorption in the UV spectrum and the efficient production of reactive species. The g-C_3_N_4_-Bi_2_S_3_ composites have significantly higher degradation rates, with g-C_3_N_4_-Bi_2_S_3_(2) attaining the maximum efficiency in UV, obtaining 90.86% degradation of para-nitrophenol within 8 h. This enhancement is due to the heterojunction between g-C_3_N_4_ and Bi_2_S_3_, which facilitates charge separation and reduces electron hole recombination. Surprisingly, Bi_2_S_3_ demonstrated no activity under UV light owing to its restricted UV responsiveness.

In visible light, the g-C_3_N_4_-Bi_2_S_3_ composites outperform both pure g-C_3_N_4_ and Bi_2_S_3_, emphasizing the contribution of Bi_2_S_3_ in broadening the light absorption range into the visible spectrum. Among the composites, g-C_3_N_4_-Bi_2_S_3_(3) has the highest performance, achieving 16.78% degradation under a visible LED source in 8 h, which is attributed to its elevated Bi_2_S_3_ concentration that improves visible light absorption and electron transfer. Pure g-C_3_N_4_ has enhanced activity under UV light but demonstrates inadequate performance in visible light, while Bi_2_S_3_ alone displays restricted activity. The findings highlight the synergistic impact of the heterojunction, enhancing photocatalytic efficiency under both UV and visible light.

The efficiency of degradation (%) of the para-nitrophenol (Table 1 and Figure S3) and the reaction kinetics were calculated using a pseudo-first-order kinetic model (Figure S4), as shown in Equations (6) and (7) in Section 3.7. Table 1 displays the percentage, the rate constant of para-nitrophenol degradation, and the R-square values for UV and Vis LEDs.

#### 2.9.2. Photoactivity Results of g-C_3_N_4_-ZnS

**Figure 9:**
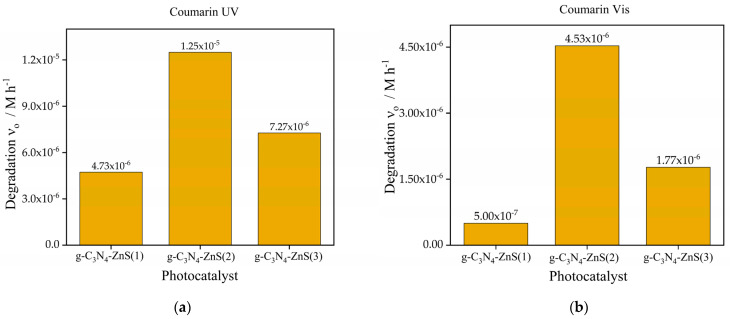
Photocatalytic degradation of coumarin (**a**) UV Source (**b**) Vis light source with photocatalyst g-C_3_N_4_-ZnS(1), g-C_3_N_4_-ZnS(2), and g-C_3_N_4_-ZnS(3).

**Figure 10:**
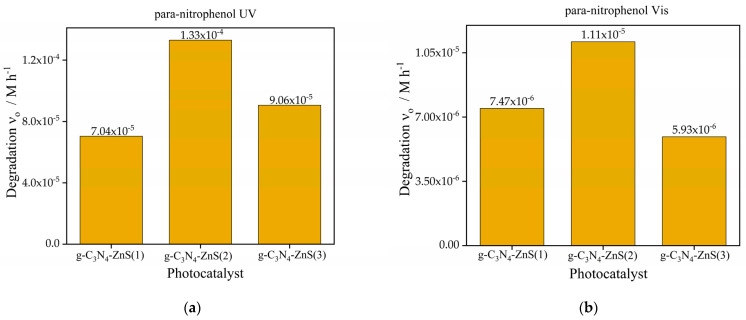
Photocatalytic degradation of para-nitrophenol (**a**) UV Source (**b**) Vis light source with photocatalyst g-C_3_N_4_-ZnS.

The initial photocatalytic degradation rates of coumarin and para-nitrophenol under UV and visible light sources utilizing g-C_3_N_4_, as depicted in Figure 6a and Figure 7a, along with three distinct zinc sulfide nanocomposites: g-C_3_N_4_-ZnS(1), g-C_3_N_4_-ZnS(2), and g-C_3_N_4_-ZnS(3), each exhibiting varying ZnS loadings, are illustrated in Figure 9a,b and Figure 10a,b.

Regarding coumarin, g-C_3_N_4_-ZnS(2) exhibits the highest degradation rate under UV light (1.25 × 10^−^^5^ M h^−1^), followed by g-C_3_N_4_-ZnS(3), and g-C_3_N_4_-ZnS(1) is shown in Figure 9a. The same pattern is seen with visible light (Figure 9b), but with diminished rates overall, likely because of the lower intensity of the visible light source (in addition to the lower excitation energy). The improved performance of g-C_3_N_4_-ZnS(2) under both UV and visible light suggests that its appropriate ZnS composition enhances charge separation and increases light utilization. The decreased activity of g-C_3_N_4_-ZnS(3) may be ascribed to an elevated concentration of ZnS nanoparticles filtering or scattering the irradiation. Notably, the band gap of ZnS is much higher than that of g-C_3_N_4_.

The initial degradation rates of para-nitrophenol are much higher than those of coumarin under both light sources. The highest degradation rate under UV light is attained by g-C_3_N_4_-ZnS(2) (1.33 × 10^−^^4^ M h^−1^), followed by g-C_3_N_4_-ZnS(3) and g-C_3_N_4_-ZnS(1). Despite the reduced overall rates in visible light, this trend persists. Due to its greater reactivity with photogenerated reactive species or its enhanced adsorption affinity on the catalyst surface, para-nitrophenol seems to degrade more rapidly than coumarin. The enhanced performance of g-C_3_N_4_- ZnS(2) is likely attributable to the superior dispersion of ZnS, which facilitates more active sites, efficient charge separation, and enhanced light absorption while minimizing electron hole recombination.

Under visible light, pure g-C_3_N_4_ showed no activity as seen in Figure 6b, but g-C_3_N_4_-ZnS composites revealed a slight enhancement in activity, with g-C_3_N_4_-ZnS(2) proving to be the most efficacious. This indicates that ZnS enhanced visible light absorption; nevertheless, the total activity remained low.

The degradation of para-nitrophenol under UV radiation, as seen in Figure 10a, exhibited a similar pattern relative to coumarin, although, in this case with higher rates than without ZnS. The g-C_3_N_4_-ZnS composites significantly surpassed pure g-C_3_N_4_ (3.8 × 10^^−^5^ M h^−1^) (Figure 7a), with g-C_3_N_4_-ZnS(2) attaining the maximum degradation rate (1.33 × 10^^−^4^ M h^−1^), followed by g-C_3_N_4_-ZnS(3) and g-C_3_N_4_-ZnS(1). The combination of ZnS with g-C_3_N_4_ improved the photocatalytic degradation of para-nitrophenol under UV illumination. The enhancement is due to the creation of a heterojunction between ZnS and g-C_3_N_4_, which promotes charge separation and enhances the availability of reactive species that contribute to the degradation process. In visible light (Figure 10b), the pure g-C_3_N_4_ exhibited negligible activity (Figure 7b), but g-C_3_N_4_-ZnS composites showed moderate performance, with g-C_3_N_4_-ZnS(2) proving to be the most effective at 1.11 × 10^^−^5^ M h^−1^. The augmented visible light activity of the composites results from the ZnS alteration, which enhances visible light absorption and facilitates the degradation process.

**Figure 11:**
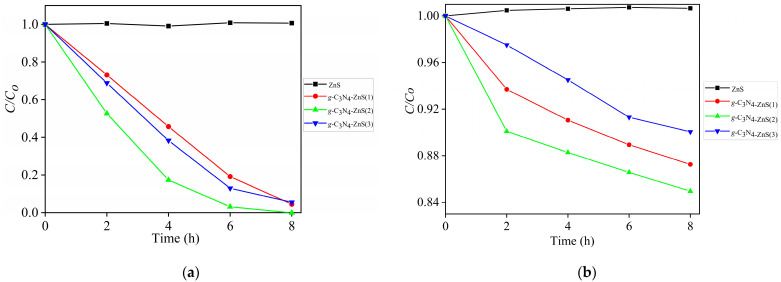
C/C_0_ curves for para-nitrophenol degradation by irradiations with g-C_3_N_4_-ZnS (**a**) UV and (**b**) visible LEDs.

Figure 11 illustrates the degradation efficiency of para-nitrophenol (C/C_0_ vs time) under UV light (a) and visible light (b) utilizing different photocatalysts. Due to excellent UV absorption and efficient reactive species production, pure g-C_3_N_4_ exhibits a modest activity under UV light (Figure 8a). The degradation rates of the g-C_3_N_4_-ZnS composites, on the other hand, are noticeably higher. In UV, g-C_3_N_4_-ZnS(2) exhibits the best efficiency, degrading 100% of para-nitrophenol in 8 h. The heterojunction between g-C_3_N_4_ and ZnS, which encourages charge separation and reduces electron hole recombination, is responsible for this enhancement. ZnS alone shows no activity under UV light owing to its large band gap.

In visible light, the g-C_3_N_4_-ZnS composites dominate both pure g-C_3_N_4_ and ZnS, demonstrating the contribution of ZnS in broadening the light absorption range into the visible spectrum. The composite with the highest performance, g-C_3_N_4_-ZnS(2), degrades 15.1% in 8 h in a Vis LED source with an optimal ZnS concentration, which improves electron transport and visible light harvesting. Pure g-C_3_N_4_ and ZnS have poor performance under visible light, but ZnS alone shows negligible activity. These findings underscore the synergistic impact of the heterojunction, enhancing photocatalytic efficiency under both UV and visible light.

Overall, the photocatalyst g-C_3_N_4_-ZnS(2) consistently demonstrates superior photocatalytic performance regardless of illumination conditions and against different pollutants. This emphasizes the need for modifying the ZnS concentration to enhance nanocomposite performance by improving charge dynamics and light absorption while inhibiting particle aggregation. The degradation rates are heightened under UV light due to the substantial UV absorption of ZnS; however, para-nitrophenol degrades more efficiently than coumarin, perhaps due to changes in structure and reactivity. These findings highlight the need for careful formulation of materials to enhance photocatalytic efficiency in various conditions. Since the band gap of ZnS (3.53 V [44]) is much higher than that of g-C_3_N_4_, it cannot function as a sensitizer. However, its conduction band potential (−1.37 V [44]) is rather close to that of g-C_3_N_4_. Hence, it can efficiently trap conduction band electrons, and, through this electron accumulation, it can both promote the generation of superoxide radicals and diminish electron-hole recombination.

Analogous to bismuth sulfide-modified catalysts, the degrading efficiency (%) of para-nitrophenol (Table 2 and Figure S5) and the kinetics of the reaction were calculated using a pseudo-first-order kinetic model (Figure S6), as shown in Equations (1) and (2) for the zinc sulfide-modified catalyst. Table 2 presents the percentage, rate constant of para-nitrophenol degradation, and the R-squared values for UV and Vis LEDs.

#### 2.9.3. Combined Photocatalytic Effect of g-C_3_N_4_-Bi_2_S_3_ and g-C_3_N_4_-ZnS

**Figure 12:**
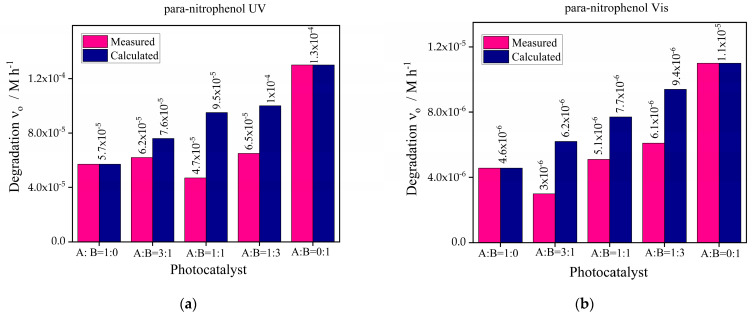
Photocatalytic degradation of para-nitrophenol (**a**) UV source (**b**) Vis light source with combined effect of photocatalysts g-C_3_N_4_-Bi_2_S_3_(2) and g-C_3_N_4_-ZnS(2).

The combined effect of the best-chosen catalysts g-C_3_N_4_-Bi_2_S_3_(2) and g-C_3_N_4_-ZnS(2) was observed under UV and visible sources with para-nitrophenol. The concentration of the catalyst in the photodegradation reaction was 1 g/L, the composition of g-C_3_N_4_-Bi_2_S_3_ and g-C_3_N_4_-ZnS was taken in the ratio (1:0, 3:1, 1:1, 1:3, 0:1). The results obtained are demonstrated in Figure 12.

Using the photocatalysts g-C_3_N_4_-Bi_2_S_3_(2), designated as A, and g-C_3_N_4_-ZnS(2), designated as B, and their combinations in ratios of A:B = 1:0, 3:1, 1:1, 1:3, and 0:1, the given graphs demonstrate the initial degradation rates of para-nitrophenol under UV and visible light. For A:B = 0:1, the best degradation rate is shown under ultraviolet (UV) light. A:B = 0:1 (pure B), which emphasizes that ZnS has exceptional UV photocatalytic activity brought forward by a desirable bandgap. Mixed ratios like A:B = 1:3, A:B = 3:1 outperform pure A, displaying synergistic interactions between A and B that improve photocatalytic efficiency by means of charge separation and supposedly a reduction in recombination inefficiencies. Higher ZnS concentration shows an increasing activity trend that indicates its major importance in UV-light-driven catalysts.

The maximum photoactivity under visible light is shown by A:B = 0:1 (pure B), demonstrating that ZnS has better photocatalytic ability in this spectrum. This result emphasizes the materials’ potential to efficiently capture visible light, most likely because of improved surface characteristics and better charge carrier dynamics. Additionally, showing substantial activity are mixtures such as A:B = 1:3 and A:B = 1:1, implying complimentary characteristics between A and B. Utilizing the advantages of both materials, pure A (A:B = 1:0) contributes less effectively but improves performance when paired with B. Even while B continuously leads the photocatalytic activity in both visible and UV light, the interaction between Bi_2_S_3_ and ZnS helps the mixed systems achieve efficient PN degradation with better light absorption and charge separation capabilities.

#### 2.9.4. Reusability

**Figure 13:**
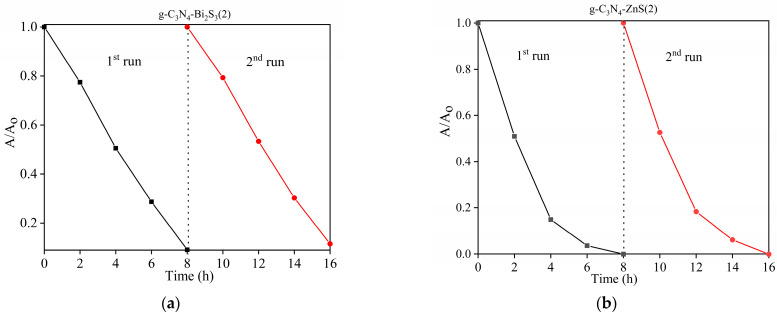
Reusability of catalysts (**a**) g-C_3_N_4_-Bi_2_S_3_(2) and (**b**) g-C_3_N_4_-ZnS(2).

The key considerations in the actual use of photocatalysis are the reliability and reusability of photocatalytic materials. The stability of g-C_3_N_4_-Bi_2_S_3_ and g-C_3_N_4_-ZnS nanocomposites was assessed using cyclic photocatalytic degradation of para-nitrophenol, as seen in Figure 13. The catalyst was centrifuged and washed with Milli-Q water after its first application. The material was allowed to dry overnight in anticipation of its second use, during which it was noted that several milligrams of catalyst were lost during the washing and centrifugation processes. According to the results of these recycling investigations, the efficiency of photocatalytic degradation was steady for around 16 h over the course of two consecutive cycles, with a little decrease that could be related to catalyst loss during reuse.

## 3. Materials and Methods

### 3.1. Materials

4-nitrophenol (C_6_H_5_NO_3_) and coumarin (C_9_H_6_O_2_) was purchased from Carlo Erba Reagents (Val-de-Reuil, France), and 1,4-hydroquinone (C_6_H_6_O_2_) ≥ 99% and 7-OH coumarin was obtained from Sigma Aldrich Kft. (Budapest, Hungary), respectively. Melamine (C_3_H_6_N_6_) was bought from Scharlab Hungary Kft. Debrecen, Hungary). Bismuth nitrate pentahydrate and zinc nitrate hexahydrade was bought from Forr-Lab Kft. (Budapest, Hungary). Starch was purchased from Reanal (Budapest, Hungary). The water used in these tests was first double-distilled and then cleaned with a Milli-Q machine (Merck Millipore, Darmstadt, Germany).

### 3.2. Synthesis of g-C_3_N_4_

A total of 10 g of precursor melamine was added to an 85 cm^3^ ceramic crucible. The crucible was kept covered and at room temperature until it was heated in an air-environment muffle furnace (a Nabertherm P330 furnace from Bartherm GmbH, Lilienthal, Germany). With a pace of 5 °C per minute up to 550 °C for 4 h, the heating process was initiated. Then, once the crucible cooled to room temperature, the product was ground into a powder and analyzed thoroughly using different instrumental methods [45].

### 3.3. Synthesis of Bismuth- and Zinc-Sulfide-Modified g-C_3_N_4_

In a typical procedure, 0.5 g of starch was mixed with 100 mL MQ water and heated to 60–70 °C under stirring for 20 min until a transparent starch solution was obtained. Bismuth nitrate was dissolved in 100 cm^3^ of hot starch solution, and it was heated to 60 °C for 10 min, under stirring conditions. Similarly, sodium sulfide (0.05 M) was dissolved in 50 cm^3^ of hot starch solution separately, and it was also heated to 60 °C for 10 min, under stirring conditions. Under hot and stirring conditions, the sodium sulfide solution was added drop wise to a bismuth–nitrate solution. The reaction mixture was heated up gently to 50 °C for 10 min, with continuous stirring. After this time, the reaction was cooled down to room temperature. The resultant black–brown colloidal suspension indicated the formation of Bi_2_S_3_ NPs [22]. The total concentration of Bi_2_S_3_ NPs was 10 mM.

A total of 300 mg of g-C_3_N_4_ was mixed with 25 cm^3^, 45 cm^3^, and 65 cm^3^ of the aforementioned Bi_2_S_3_ NPs solution individually and stirred overnight. The resulting compound was then dried in an oven at 60 °C overnight and used for photocatalytic applications. It was designated as g-C_3_N_4_-Bi_2_S_3_(1), g-C_3_N_4_-Bi_2_S_3_(2), an d g-C_3_N_4_-Bi_2_S_3_(3). The same technique was used for the synthesis of ZnS nanoparticles, resulting in a milky white colloidal solution that signifies the synthesis of zinc sulfide. Also in this case, the total concentration of ZnS NPs was 10 mM. Additionally, a process similar to that of g-C_3_N_4_-Bi_2_S_3_ was used for the synthesis of g-C_3_N_4_-ZnS, designated as g-C_3_N_4_-ZnS(1), g-C_3_N_4_-ZnS(2), and g-C_3_N_4_-ZnS(3).

Pure solid Bi_2_S_3_ and ZnS were also synthesized by direct addition of sodium sulfide (0.05 M) to metal nitrate solution without using starch.

### 3.4. ICP Measurements

#### 3.4.1. Sample Preparation for ICP-OES Analysis

The microwave acid digestion was applied for sampled dissolution and was carried out in closed vessel. Anton Paar Multiwave 3000 type device (Anton Paar GmbH, Graz, Austria) was used with microwave energy of 600 W. The sample was weighted to the nearest 0.1300 g into a fluorocarbon polymer microwave vessel (HF100), followed by the addition of an aqua reqia solution in an amount of 6 mL (4.5 cm^3^ HCl + 1.5 cm^3^ HNO_3_). The vessel was sealed and heated in the microwave unit equipped with pressure and temperature controllers. The temperature was ramped to 175 °C within 30 min followed by a contact time of 30 min. Then, the vessels were cooled, vented, and opened. After cooling, the total dissolved sample content was diluted to volume of 25 cm^3^ and the samples were analyzed by the ICP-OES method.

#### 3.4.2. Analytical Analysis

Elemental analysis was performed using inductively coupled plasma optical emission spectrometry (ICP-OES) with PerkinElmer Avio Max 550 instrument (PerkinElmer Inc., Waltham, MA, USA). The multi-element standard solutions (Supelco standard of 1000 mg/L for Bi, Zn and TruQ standard 100 mg dm^−3^ for S) were diluted to produce calibration solutions for the investigated elements (0, 0.05, 0.01, and 1 mg dm^−3^). The wavelengths of the identification lines were as follows: Bi 223.065 nm, Zn 206.200 nm, and S 181.975 nm. Each sample was measured three times, and the relative standard deviations (RSD) for investigated metals was less than 5%.

### 3.5. Characterization of Catalyst

A Philips PW 3710 powder diffractometer (Philips Analytical B.V., Almelo, The Netherlands) was used to acquire the X-ray diffraction patterns. A copper K-alpha radiation source with a wavelength of 1.5405 Å was installed in the diffractometer.

To carry out scanning electron microscopy observations, the Philips/FEI XL 30 environmental scanning electron microscope (Philips/FEI, Hillsboro, OR, USA) was used. When imaging, the Apreo SEM (ThermoFisher Apreo S, FEI/ThermoFisher, Waltham, MA, USA) scanning electron microscope) equipped with Octane Elect Plus EDS (AMETEK, Inc., Brewyn, PA, USA) was used at a voltage of 5.0 kV, while the elemental mapping was performed at a voltage of 25.0 kV.

The infrared spectra of nanocomposite samples were obtained using a Bruker Vertex 70 Fourier-transform infrared (FTIR) spectrometer (Bruker GmbH, Rosenheim, Germany) fitted with a Bruker Diamond ATR sample chamber, operating at a resolution of 2 cm^−1^ with a room temperature DTGS detector. The final spectra were obtained by averaging 512 images with atmospheric adjustment.

The size and shape of the g-C_3_N_4_-Bi_2_S_3_ and g-C_3_N_4_-ZnS nanocomposite were examined using transmission electron microscopy (TEM). Cu grids covered with a fine covering of a lacey carbon sheet were subjected to the suspension. At room temperature, the grids were left to air dry. An FEI Talos F200X G2 (Thermo Fisher Scientific, Brno, Czech Republik) scanning transmission electron microscope (STEM) was then used to analyze the grids. Bright-field (BF) images, selected-area electron diffraction (SAED) patterns, and high-resolution transmission electron microscopy (HRTEM) images were utilized to examine the two-dimensional morphology of g-C_3_N_4_ and its incorporation of bismuth sulfide and zinc sulfide nanoparticles. The microscope was utilized in TEM mode with a beam current of around 80 pA and an accelerating voltage of 200 kV. Using STEM spectrum imaging mode, Energy-Dispersive X-ray (EDX) spectra were obtained for each pixel inside the image to acquire elemental maps. Energy-Dispersive X-ray (EDX) studies and elemental mapping were performed using a four-detector SuperX silicon drift detector system. The beam current was around 200 pA while the STEM mode was operating. Velox 2.14 software was used to evaluate the collected data.

The N_2_ adsorption–desorption isotherms at −196 °C determined the specific surface area, pore volume, and pore size distribution in the 1.7–100 nm diameter range. Degassing was performed using a Micromeritics FlowPrep 060-type (Micromeritics Instrument Corp., Norcross, GA, USA) at 160 °C for 2 h at a static condition before transferring to the Micromeritics 3Flex 3500-type instrument (Micromeritics Instrument Corp., Norcross, GA, USA) and continuing under vacuum for 4 h. The particular area was calculated using Brunauer–Emmett–Teller (BET). T-plots presented Smicro and Vmicro data. The Barrett–Joyner–Halenda (BJH) model was used to compute mesopore size distributions, total pore volume (V), and average pore diameter (Dav) using nitrogen desorption isotherms.

The chemical state and elements found on the sample surfaces were examined using a Thermo Scientific ESCALAB Xi^+^ instrument (Thermo Fisher Scientific, Brno, Czech Republik). A 650 µm spot size monochromatized Al K-alpha source (1486.6 eV) was used. The pressure in the analysis room was less than 10^−9^ mbar prior to the trial. To gain a comprehensive understanding of the elements present, a large number of distinct spectra were obtained from each sample (at an analyzer pass energy of 80 eV). To measure and investigate the chemical state, high-resolution spectra (at 40 eV pass energy) of the photoelectron lines for C 1s, N 1s, O 1s, Bi 4f, S 2p, S 2s, and Zn 2p areas were obtained. The automated built-in charge adjustment mechanism compensated for the surface charge of the sample. In order to fine-tune the energy scale, the energy of sp^2^-bonded C in N=C(–N)_2_ was set at 288.2 eV.

The diffuse reflectance spectra were measured using a Specord S600 spectrofluorometer with an integrating sphere (Analytik Jena GmbH +Co., Jena, Germany), and the Tauc method was used to calculate the band-gap values.

### 3.6. Investigation of Photocatalytic Activity

A 70 cm^3^ laboratory-scale quartz reactor was used for photocatalytic tests. The visible LEDs used had a maximum wavelength of 453 nm and a power of 14 W (2 × 7 W), while the UV LED had a maximum wavelength of 374 nm, and a power of 50 W. Figure S7 displays the emission spectra. The light sources were positioned 10 cm apart on each side of the reactor. Air bubbles were introduced to the reaction mixture at a flow rate of 10 dm^3^ h^−1^ throughout all experimental trials to agitate it. The degradation of colorless organic molecules in an aqueous solution was used to measure the performance of g-C_3_N_4_-Bi_2_S_3_ and g-C_3_N_4_-ZnS nanocomposites in photocatalysis. To do this, we compared the solution spectrum absorbance before and after exposure to ultraviolet (UV) and visible (Vis) light from LEDs; Figure S8 shows the arrangements for both types of irradiations. Despite a little rise of 1–2 °C that had no effect on adsorption, desorption, or the photochemical reaction, the reaction mixture temperature was rather constant under illumination. The experimental approach included adding 50 mg of catalyst to 5 cm^3^ of Milli-Q water, followed by a 10 min sonication operation. After adding 45 cm^3^ of model compound and stirring continuously for 30 min in the dark, we achieved particle homogeneity and reached adsorption–desorption equilibrium in the suspension (with a catalyst concentration of 1 g dm^−3^). Subsequently, either the visible or UV LEDs were turned on, samples were collected using a 4-cm^3^ syringe at different intervals, and a Millipore Millex-LCR PTFE (Millex, Darmstadt, Germany) 0.45 µm membrane filter was used for filtering.

### 3.7. Model Compounds

During the irradiation of the photocatalyst, emissive 7-hydroxycoumarin (7-OHC) was created by directly interacting with OH radicals, commencing with a concentration of 9 × 10^−5^ M of coumarin. After quantifying the absorbances of coumarin at 277 nm (11,308 M^−1^ cm^−1^), the change in concentration of coumarin was determined using a Scinco S-3100 UV–Vis spectrophotometer (Scinco Co. Ltd., Seoul, Republic of Korea). At 277 nm, the 7-OHC compound had a much lower molar absorption coefficient (ε) of 3209 M^−1^ cm^−1^ compared to the coumarin compound. Light absorption was, therefore, unaffected by the produced 7-OHC at low quantities. Measurements of 7-OHC emission (λ_excitation_ = 332, λ_emission_ = 453 nm) were taken using a spectrofluorometer (PerkinElmer LS50B). Calibration curves were used to estimate the amounts of the residual coumarin, and the 7-OHC that was generated (Figure S9a,b). Plots of the emission intensity values at 453 nm vs. 7-OHC concentration and of the absorbance at 277 nm vs. coumarin concentration were plotted.

The concentration of para-nitrophenol was 5 × 10^−4^ M, and the absorbance spectra revealed that it peaked at 318 nm. In order to investigate the optical characteristics, a UV-visible absorption spectroscopy experiment was carried out using a 0.2 cm^3^ cuvette. Figure S10 shows the calibration curve, and the intermediate formed during irradiation was used to compute the change in para-nitrophenol concentration.

The efficiency of degradation of the para-nitrophenol and coumarin (D(t), %) was determined using Equation (6):

Degradation efficacy = (C_0_ − C_t_/C_0_) × 100 (6)

The reaction’s kinetics were predicted using a pseudo-first-order kinetic model, as shown in Equation (7):

−ln (C_t_/ C_0_) = *k*t (7)

where C_0_ and C_t_ are the initial concentration and the final concentration of organic pollutants at time t (h), respectively, and *k* (h^−1^) is the rate constant.

## 4. Conclusions

The g-C_3_N_4_-Bi_2_S_3_ and g-C_3_N_4_-ZnS composites were effectively synthesized by the application of a starch-assisted method. The Bi_2_S_3_ and ZnS nanoparticles are uniformly distributed and strongly attached to the surface of g-C_3_N_4_, minimizing substantial agglomeration owing to the presence of starch, which serves as a stabilizing factor. The composite absorbs both UV and visible light radiation, as shown by the UV–vis DRS spectra. The photodegradation results clearly demonstrated that the enhanced photocatalytic activity of g-C_3_N_4_-Bi_2_S_3_ and g-C_3_N_4_-ZnS composites was primarily due to their improved photocarrier separation and transfer rates. Although a considerable fraction of Bi and Zn existed at the surface of the g-C_3_N_4_ particles as oxides, their sulfides primarily resulted in the improvement of photocatalytic efficiency. Our results highlight the crucial function of starch in forming heterojunctions contributing to the increased activity of g-C_3_N_4_-Bi_2_S_3_ and g-C_3_N_4_-ZnS composites. This study enhanced the green synthesis techniques for nanocomposites while also delivering significant insights into the photocatalytic degradation of para-nitrophenol, presenting prospective applications for mitigating environmental pollution issues.

## Figures and Tables

**Table 1:** Percentage of para-nitrophenol degradation, rate constant, and R-square values with bismuth sulfide modified catalyst.

Catalyst	% Degrad.		k		R^2^	
	(8 h)		(h^−1^)			
	at Irradiation with		at Irradiation with		at Irradiation with	
	UV	Vis	UV	Vis	UV	Vis
g-C_3_N_4_	59	0	1.1 × 10^−1^	0	0.98	0
g-C_3_N_4_-Bi_2_S_3_(1)	88.07	7.4	2.6 × 10^−1^	9.8 × 10^−3^	0.91	0.99
g-C_3_N_4_-Bi_2_S_3_(2)	90.86	11.7	2.9 × 10^−1^	1.6 × 10^−2^	0.92	0.99
g-C_3_N_4_-Bi_2_S_3_(3)	87.4	16.78	2.5 × 10^−1^	2.2 × 10^−2^	0.95	0.99

**Table 2:** Percentage, rate constant of para-nitrophenol degradation, and the R-squared values of zinc-modified catalysts in UV and Vis sources.

Catalyst	% Degrad.		k		R^2^	
	(8 h)		(h^−1^)			
	at Irradiation with		at Irradition with		at Irradiation with	
	UV	Vis	UV	Vis	UV	Vis
g-C_3_N_4_	59	0	1.1 × 10^−1^	0	0.98	0
g-C_3_N_4_-ZnS(1)	95.5	12.7	3.7 × 10^−1^	1.6 × 10^−2^	0.92	0.93
g-C_3_N_4_-ZnS(2)	100	15.1	5.7 × 10^−1^	1.8 × 10^−2^	0.96	0.82
g-C_3_N_4_-ZnS(3)	94.5	9.9	3.7 × 10^−1^	1.3 × 10^−2^	0.97	0.98

## Data Availability

The data presented in this study are available on request from the corresponding author. The data are not publicly available due to privacy.

## Supplementary Materials

The following supporting information can be downloaded at: https://www.mdpi.com/article/10.3390/molecules30020253/s1, Figure S1: FTIR spectra of ZnS and Bi_2_S_3_; Table S1: Bi and Zn contents of the composite catalysts; Table S2: BET surface area of the catalyst samples; Table S3: Atomic weight % values determined by XPS and EDX measurements; Figure S2: Tauc plots for determination of band-gap energy; Table S4: Band-gap energies of photocatalysts; Figure S3: Degradation efficiency of bismuth sulfide-modified catalyst under UV and Vis LED irradiation; Figure S4: Pseudo-first-order kinetic model for the bismuth sulfide-modified catalyst under UV and LED irradiation; Figure S5: Degradation efficiency of zinc sulfide-modified catalyst under UV and Vis LED irradiation; Figure S6: Pseudo-first-order kinetic model for the zinc sulfide-modified catalyst under UV and LED irradiation; Figure S7: Emission spectra of UV (black) and Vis (red) light sources applied; Figure S8: Lab-scale quartz glass reactors and their arrangement in the setup for photocatalytic experiments under (A) UV and (B) visible light; Figure S9: Calibration curve of (a) coumarin and (b) 7-OH coumarin; Figure S10: Calibration curve of para-nitrophenol.
