# Supplementary material for: g-C3N4 Modified with Metal Sulfides for Visible-Light-Driven Photocatalytic Degradation of Organic Pollutants
Source: Molecules. 2025 Jan 10;30(2):253. doi: 10.3390/molecules30020253 (PMC11767285; doi:10.3390/molecules30020253)
Supplement: Supplementary file 1 [file molecules-30-00253-s001.zip › molecules-3374483-supplementary.pdf]

Supplementary Materials

for

g-C_3N_4 modified with metal sulfides for visible-light-driven photocatalytic degradation of organic pollutants

Shoaib Mukhtar [1], Erzsébet Szabó-Bárdos [1], Csilla Őze [2], Tatjána Juzsakova [3], Kornél Rácz [4,5], Miklós Németh [6] and Ottó Horváth [1,*]

1 Research Group of Environmental and Inorganic Photochemistry, Center for Natural Sciences, Faculty of Engineering, University of Pannonia, P.O. Box 158, H-8201 Veszprém, Hungary; mukhtar.shoaib@phd.mk.uni-pannon.hu (S.M.),szabone.bardos.erzsebet@mk.uni-pannon.hu (E.S.-B.); horvath.otto@mk.uni-pannon.hu (O.H.)
2 Department of Materials Engineering, Research Center for Engineering Sciences, University of Pannonia, H-8201 Veszprém, POB. 158, Hungary; oze.csilla@mk.uni-pannon.hu
3 Sustainability Solutions Research Lab, Research Center for Biochemical, Environmental and Chemical Engineering, University of Pannonia, H-8201 Veszprém, POB. 158, Hungary; juzsakova.tatjana@mk.uni-pannon.hu
4 Nanolab, Research Institute of Biomolecular and Chemical Engineering, University of Pannonia, H-8201 Veszprém, POB. 158, Hungary; racz.kornel@mk.uni-pannon.hu
5 HUN-REN_PE Environmental Mineralogy Research Group, H-8201 Veszprém, POB. 158, Hungary; racz.kornel@mk.uni-pannon.hu
6 Centre for Energy Research, Surface Chemistry and Catalysis Department, Konkoly-Thege Street 29-33, H-1121 Budapest, Hungary; nemeth.miklos@ek-cer.hu
* Correspondence: horvath.otto@mk.uni-pannon.hu (O.H.); Tel: +36-88-624-000 / 6049 ext.

Content **Page Nr.**

Figure S1,Table S1 2

Table S2,3 3

Figure S2, Table S4 4

Figures S3,4 5

Figures S5,6 6

Figures S7,8 7

Figure S9,10 8

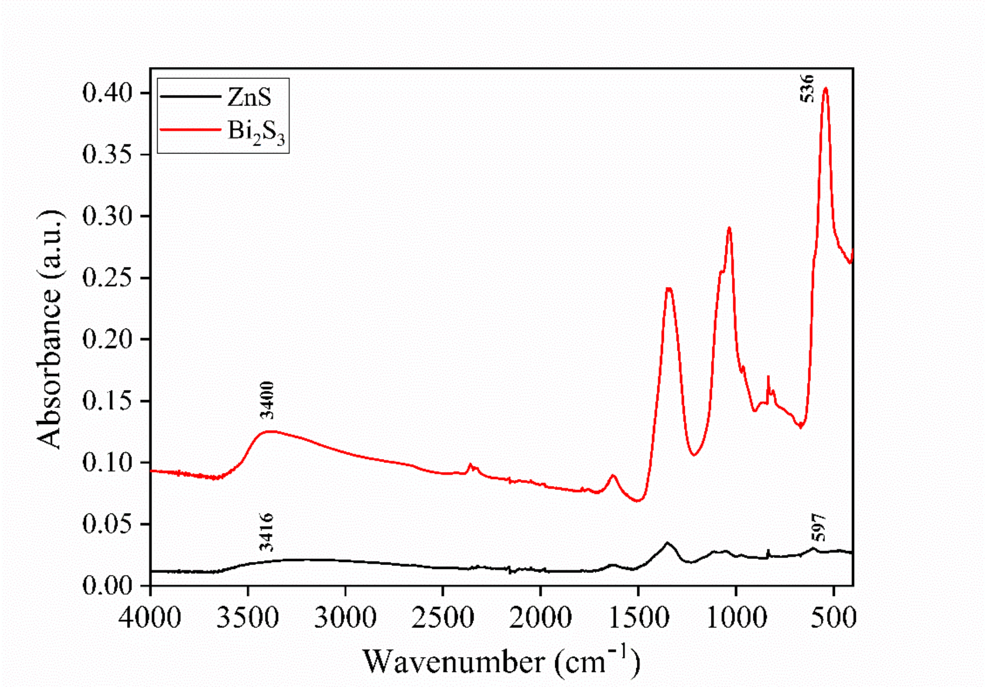

Figure S1. FTIR spectra of ZnS and Bi_2S_3.

Table S1. Bi and Zn contents of the composite catalysts.

	**g-C_3N_4-Bi_2S_3(1)**	**g-C_3N_4-Bi_2S_3(2)**	**g-C_3N_4-Bi_2S_3(3)**
**Bi (w%)**	1.87	3.41	4.13
**S (w%)**	0.1	0.18	0.2
**Bi_2S_3 (w%)**	0.54	0.96	1.07
**$Bi_2(CO_3)O_2$ (w%)**	1.75	3.2	3.98
**Bi in sulfide form (mol%)**	23.2	22.98	21.08
	**g-C_3N_4-ZnS(1)**	**g-C_3N_4-ZnS(2)**	**g-C_3N_4-ZnS(3)**
**Zn (w%)**	3.99	5.01	6.4
**S (w%)**	0.55	0.6	0.9
**ZnS (w%)**	1.76	1.82	2.74
**ZnO (w%)**	3.57	4.71	6.67
**Zn in sulfide form (mol%)**	28.17	24.47	28.74

Table S2. BET surface area of the catalyst samples.

SN	Catalyst	S_{BET} (m^2/g)	V (cm^3/g)	D_{av} (nm)
**1**	g-C_3N_4	27.9	0.19479	24.3
**2**	g-C_3N_4-Bi_2S_3(1)	12.9	0.09894	29.1
**3**	g-C_3N_4-Bi_2S_3(2)	8.9	0.06227	25.0
**4**	g-C_3N_4-Bi_2S_3(3)	6.9	0.05133	26.5
**5**	g-C_3N_4-ZnS(1)	4.2	0.03735	35.1
**6**	g-C_3N_4-ZnS(2)	2.6	0.02627	34.6
**7**	g-C_3N_4-ZnS(3)	2.9	0.02752	37.2

Table S3. Atomic weight % values determined by XPS and EDX measurements.

**g-C_3N_4**								
	C	N	O	S	Bi	Zn	Na	Si
EDX	47.39	43.9	8.71					
XPS	41.45	57.76	0.79					
**g-C_3N_4-Bi_2S_3(1)**								
	C	N	O	S	Bi	Zn	Na	Si
EDX	39.52	33.61	26.46	0.04	0.02		0.28	0.07
XPS	49.97	26.77	22.79	0.14	0.03		0.30	
**g-C_3N_4-Bi_2S_3(2)**								
	C	N	O	S	Bi	Zn	Na	Si
EDX	41.11	37.83	20.48	0.09	0.04		0.44	0.01
XPS	44.02	44.67	10.62	0.27	0.13		0.30	
**g-C_3N_4-Bi_2S_3(3)**								
	C	N	O	S	Bi	Zn	Na	Si
EDX	41.11	27.09	30.97	0.11	0.06		0.59	0.06
XPS	51.98	19.22	27.08	0.27	0.1		1.35	
**g-C_3N_4-ZnS(1)**								
	C	N	O	S	Bi	Zn	Na	Si
EDX	39.24	52.13	8.34	0.11		0.16		0.01
XPS	44.48	36.87	15.38	0.73		1.75	0.79	

g-C_3N_4-ZnS(2)								
	C	N	O	S	Bi	Zn	Na	Si
EDX	36.55	39.63	22.87	0.35		0.59		0.02
XPS	41.70	36.82	16.21	0.97		2.48	1.82	
**g-C_3N_4-ZnS(3)**								
	C	N	O	S	Bi	Zn	Na	Si
EDX	38.24	38.04	22.72	0.36		0.61		0.03
XPS	42.46	35.6	16.74	1.00		2.40	1.81	

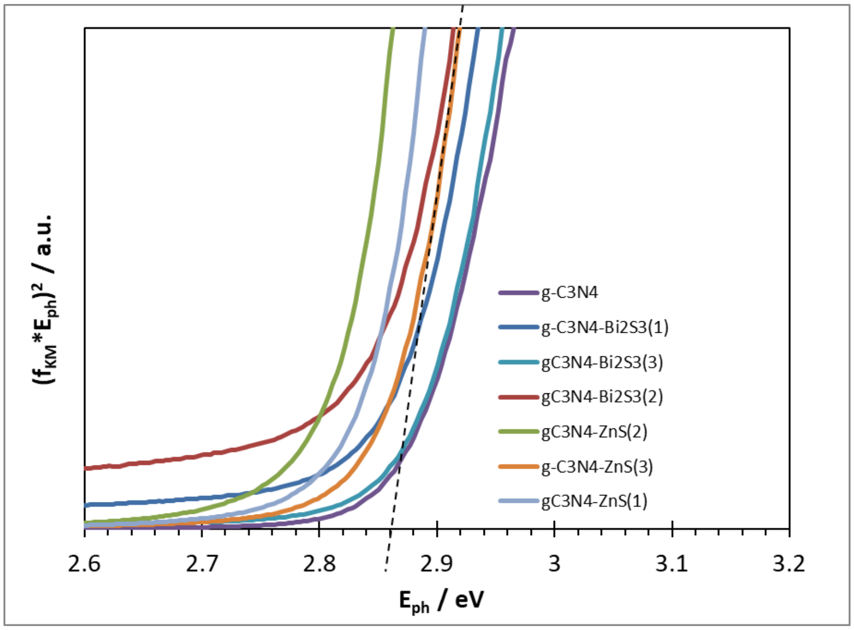

Figure S2. Tauc plots for determination of band-gap energy.

Catalyst	Band gap energy (eV)
g-C_3N_4	2.88
g-C_3N_4-Bi_2S_3(1)	2.86
g-C_3N_4-Bi_2S_3(2)	2.84
g-C_3N_4-Bi_2S_3(3)	2.88
g-C_3N_4-ZnS(1)	2.84
g-C_3N_4-ZnS(2)	2.81
g-C_3N_4-ZnS(3)	2.86

Table S4. Band-gap energies of photocatalysts.

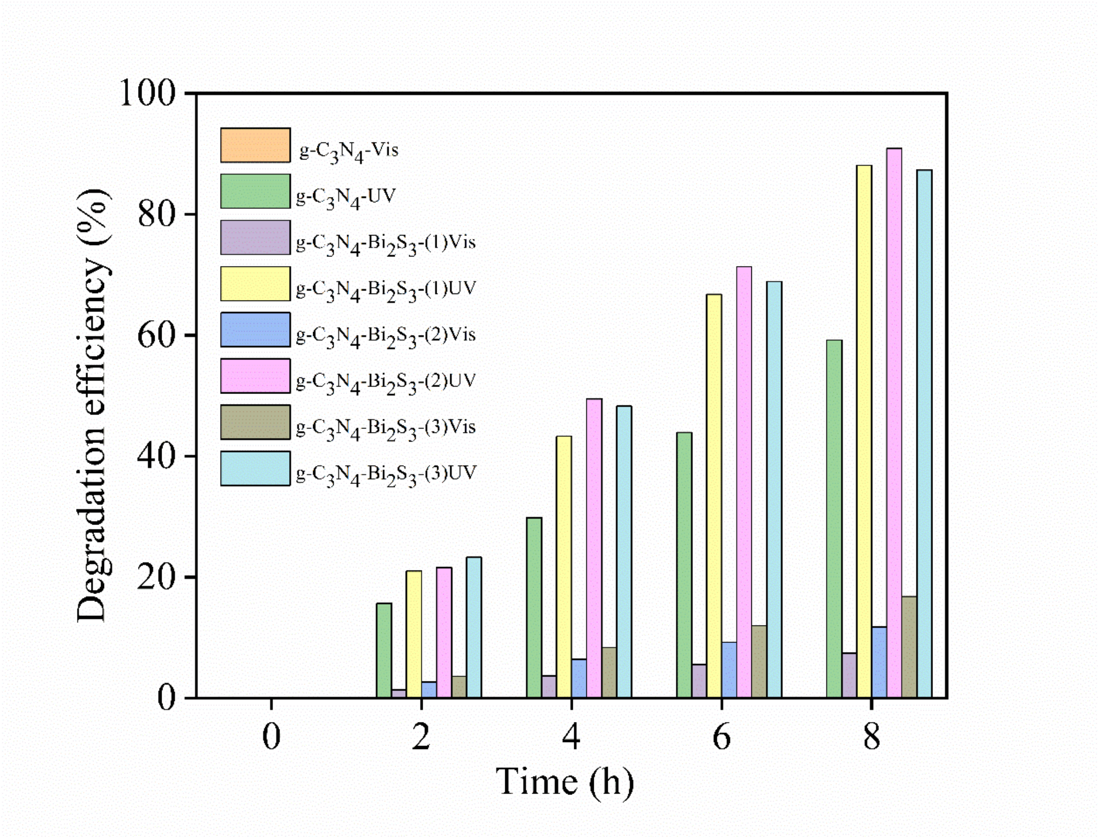

Figure S3. Degradation efficiency of bismuth sulfide-modified catalyst under UV and Vis LED irradiation.

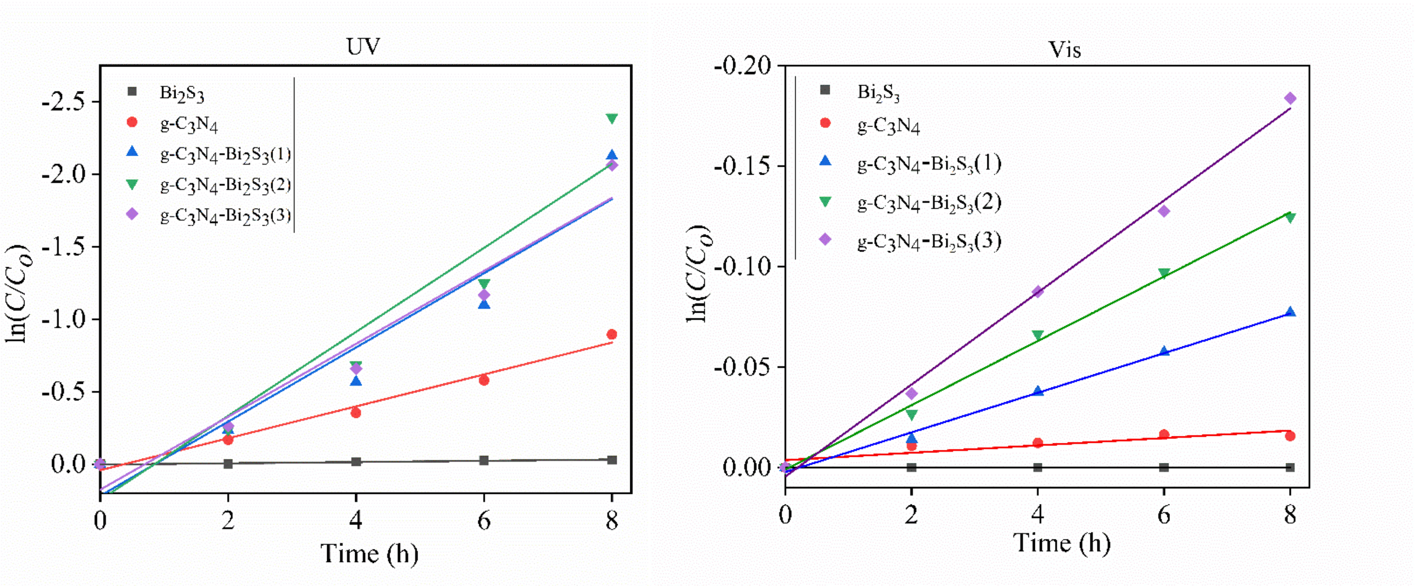

Figure S4. Pseudo-first-order kinetic model for the bismuth sulfide-modified catalyst under UV and LED irradiation.

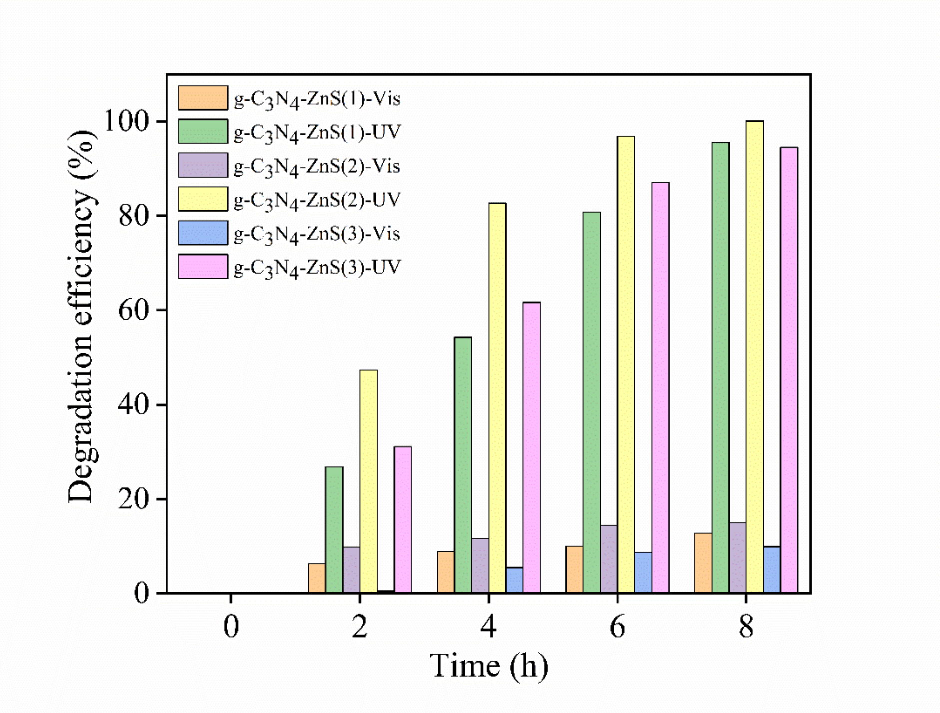

Figure S5. Degradation efficiency of zinc sulfide-modified catalyst under UV and Vis LED irradiation.

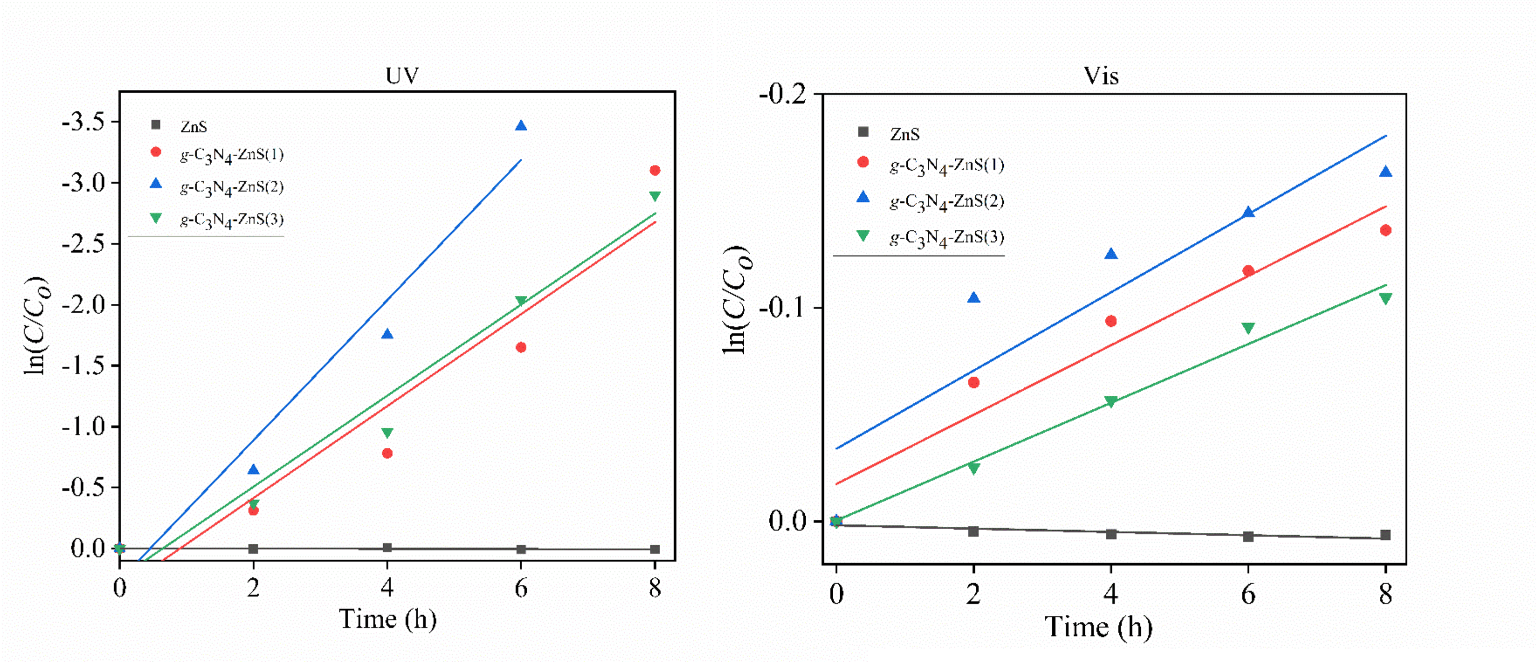

Figure S6. Pseudo-first-order kinetic model for the zinc sulfide-modified catalyst under UV- and Vis LED irradiation.

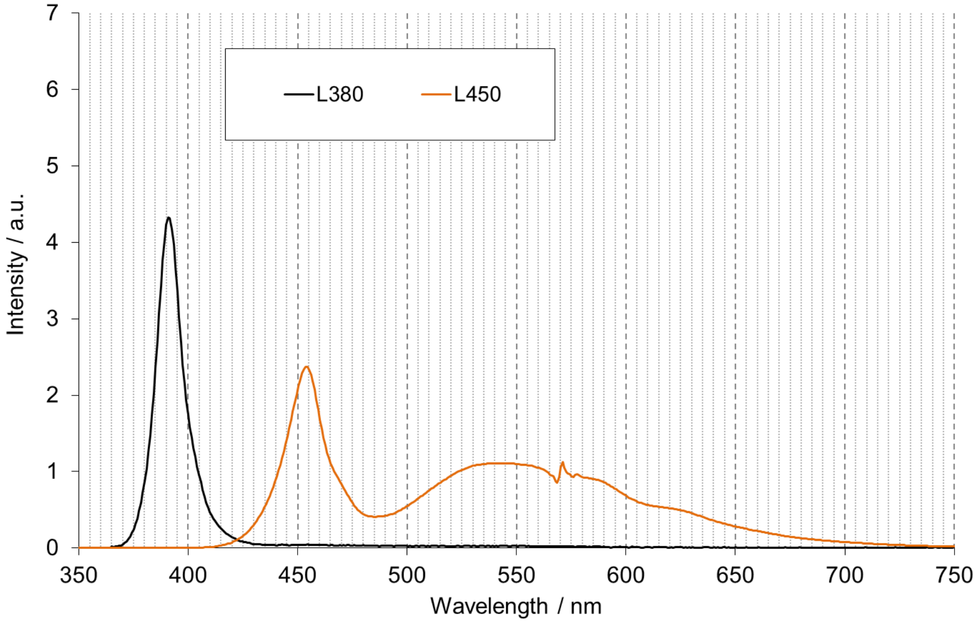

Figure S7. Emission spectra of UV (black) and Vis (red) light sources applied.

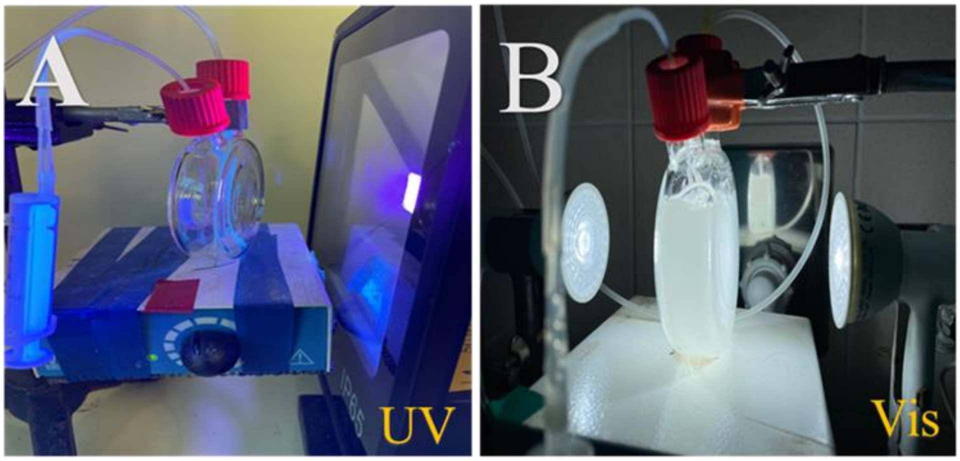

Figure S8. Lab-scale quartz glass reactors and their arrangement in the setup for photocatalytic experiments under (A) UV and (B) visible light.

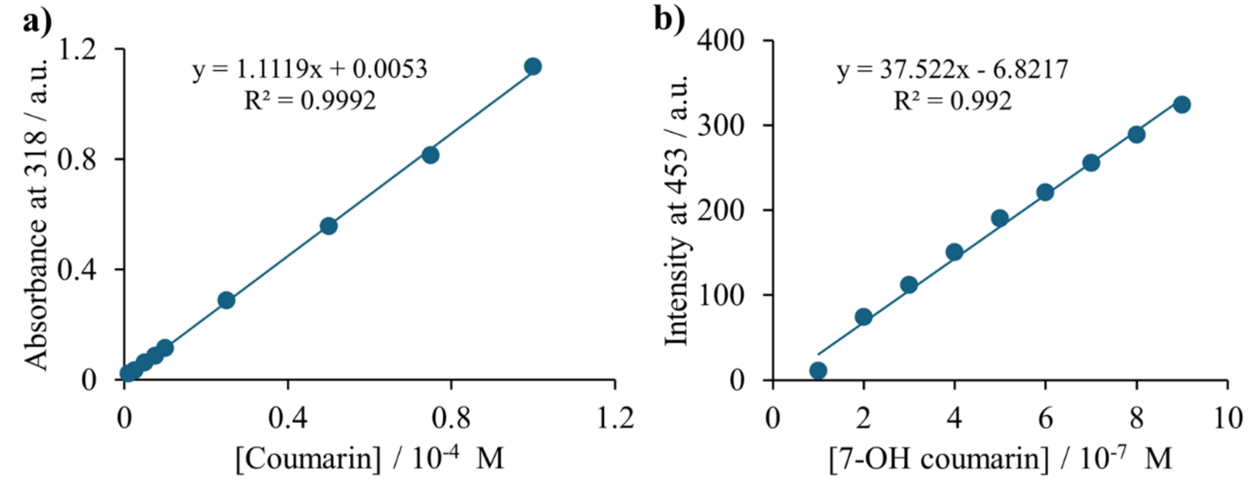

Figure S9. Calibration curve of (a) coumarin and (b) 7-OH coumarin.

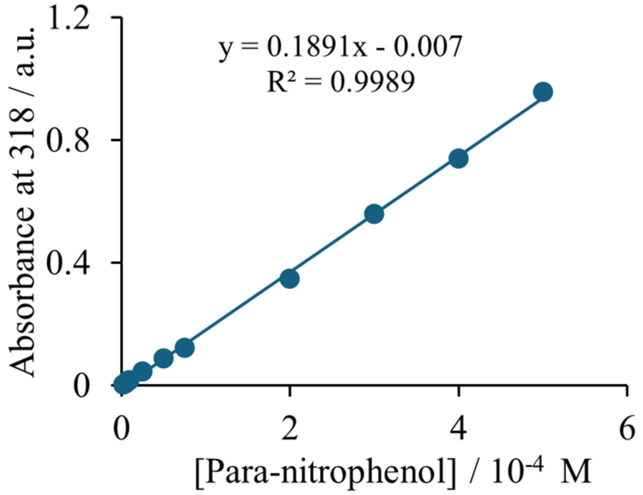

Figure S10. Calibration curve of para-nitrophenol.
